# Profile and functional analysis of small RNAs derived from *Aspergillus fumigatus* infected with double-stranded RNA mycoviruses

**DOI:** 10.1186/s12864-017-3773-8

**Published:** 2017-05-30

**Authors:** Selin Özkan, Irina Mohorianu, Ping Xu, Tamas Dalmay, Robert H. A. Coutts

**Affiliations:** 1 Department of Life Sciences, Faculty of Natural Sciences, Imperial College London, London, UK; 2 School of Biological Sciences, University of East Anglia, Norwich, UK; 3 Current Address: Vocational School of Health Services, Ahi Evran University, Kırşehir, Turkey; 4 Current Address: Geography, Environment and Agriculture Division, Department of Biological and Environmental Sciences, School of Life and Medical Sciences, University of Hertfordshire, Hatfield, UK; 5 School of Computing Sciences, University of East Anglia, Norwich, UK

**Keywords:** *Aspergillus fumigatus*, sRNA-seq, miRNA-like sRNA, Differential expression, Double-stranded RNA mycovirus, vsiRNA, Fungal sRNA

## Abstract

**Background:**

Mycoviruses are viruses that naturally infect and replicate in fungi. *Aspergillus fumigatus,* an opportunistic pathogen causing fungal lung diseases in humans and animals, was recently shown to harbour several different types of mycoviruses. A well-characterised defence against virus infection is RNA silencing. The *A. fumigatus* genome encodes essential components of the RNA silencing machinery, including Dicer, Argonaute and RNA-dependent RNA polymerase (RdRP) homologues. Active silencing of double-stranded (ds)RNA and the generation of small RNAs (sRNAs) has been shown for several mycoviruses and it is anticipated that a similar mechanism will be activated in *A. fumigatus* isolates infected with mycoviruses.

**Results:**

To investigate the existence and nature of *A. fumigatus* sRNAs, sRNA-seq libraries of virus-free and virus-infected isolates were created using Scriptminer adapters and compared. Three dsRNA viruses were investigated: Aspergillus fumigatus partitivirus-1 (AfuPV-1, PV), Aspergillus fumigatus chrysovirus (AfuCV, CV) and Aspergillus fumigatus tetramycovirus-1 (AfuTmV-1, NK) which were selected because they induce phenotypic changes such as coloration and sectoring. The dsRNAs of all three viruses, which included two conventionally encapsidated ones PV and CV and one unencapsidated example NK, were silenced and yielded characteristic vsiRNAs together with co-incidental silencing of host fungal genes which shared sequence homology with the viral genomes.

**Conclusions:**

Virus-derived sRNAs were detected and characterised in the presence of virus infection. Differentially expressed *A. fumigatus* microRNA-like (miRNA-like) sRNAs and small interfering RNAs (siRNAs) were detected and validated. Host sRNA loci which were differentially expressed as a result of virus infection were also identified. To our knowledge, this is the first study reporting the sRNA profiles of *A. fumigatus* isolates.

**Electronic supplementary material:**

The online version of this article (doi:10.1186/s12864-017-3773-8) contains supplementary material, which is available to authorized users.

## Background

Aspergillus is a mould fungus which occurs worldwide in decaying vegetation, seeds and grains. *Aspergillus fumigatus* can infect humans, causing life-threatening opportunistic infections in immunocompromised individuals including transplant, AIDS and leukaemia patients, as well as being the causative agent of allergic diseases [32, 46]. *A. fumigatus* virulence is multifactorial and is a consequence of its structure (such as cell wall), growth capacity and its ability to damage its host [1].

Mycovirus infection in *A. fumigatus* was initially investigated in 61 isolates and revealed that apparently none contained dsRNA elements [57]. However, in a subsequent study, at least three different dsRNA profiles were observed following screening of more than 360 *A. fumigatus* isolates [12]. Three mycoviruses, Aspergillus fumigatus chrysovirus (AfuCV; [27]), Aspergillus fumigatus partitivirus (AfuPV-1; [10]) and Aspergillus fumigatus tetramycovirus*-*1 (AfuTmV-1; [30]) have since been fully characterised and sequenced. In another recent investigation, several different dsRNAs were discovered in a Dutch collection of *A. fumigatus* isolates [49] none of which had any significant similarity to the ones described in the current investigation.

RNA silencing is an internal defence mechanism which protects genomes against invasion by mobile genetic elements such as viruses and transposons [2]. Virus-derived small interfering siRNAs (vsiRNAs) are generated as a result of recognition and processing of viral dsRNA by Dicer enzymes and have been described in many organisms harbouring viruses including animals and plants [21, 62, 63]. In plants, where post-transcriptional gene silencing (PTGS) acts as an antiviral defence, the first evidence of a role of RNA interference (RNAi) in virus defence was shown in a natural, virus-induced RNA silencing model [48]. The trigger for RNA silencing is dsRNA; in plants viral siRNAs were found to be derived predominantly from single stranded RNA (ssRNA) viruses following RdRP activity which converts ssRNA into dsRNA [39]. Moreover, it was also shown that plants gain resistance against DNA viruses through siRNA-mediated methylation [53]. The majority of these studies were performed with viruses that possess an ssRNA genome, however viruses with dsRNA genomes are of particular interest as they are both triggers and targets of the host RNA silencing machinery [15, 17, 25, 60]. In animals, especially insects, vsiRNAs are generated from viral dsRNAs processed by the RNA silencing machinery [42, 62].

The antiviral role of the RNA silencing machinery has also been described in fungi. It was first observed in the chestnut blight fungus *Cryphonectria parasitica* where virus infection cannot be eliminated and viral RNA levels increased in mutants of RNA silencing components. It was reported that in the presence of *Cryphonectria* hypovirus*-*1 (CHV-1) (an ssRNA virus) a *C. parasitica* Dicer mutant was debilitated as compared to the wild type [52]. Furthermore, the presence of mycovirus-derived siRNAs in *C. parasitica*, [64], *Magnaporthe oryzae* [27] and significantly *Aspergillus nidulans* [25] demonstrated the existence of active antiviral defence in filamentous fungi. In another investigation the effects of four different *C. parasitica* hypoviruses on RNA silencing using Dicer mutants revealed that silencing responses differed for each virus irrespective of the fact that the proteins encoded by the viruses were 90–99% identical at the amino acid sequence level [65]. Subsequently numerous examples of silencing dsRNAs from several different mycovirus families including victoriviruses [18, 60], partitiviruses [55], quadriviruses [60] and an unassigned mycovirus of *Colletotrichum higginsianum* [15], have all been reported. These findings are significant since it was thought previously that the genomes of these encapsidated mycoviruses were not available as templates for silencing [60]. Mycoviruses have been considered as potential biological control agents as they can induce phenotypic alterations through RNA silencing in different ways. For instance significant complementarity between - mycovirus-derived siRNAs and host gene sequences can induce changes in gene expression [25, 58].

In this paper we investigated *A. fumigatus* sRNAomes in the presence and absence of three mycoviruses: Aspergillus fumigatus partitivirus-1 (AfuPV-1, PV), Aspergillus fumigatus chrysovirus (AfuCV, CV) and Aspergillus fumigatus tetramycovirus-1 (AfuTmV-1, NK). Both AfuPV and AfuCV cause visible phenotypic alterations and result in a decreased growth rate as compared to virus-free isolates; however they had no impact on fungal pathogenicity as assessed in the murine model [11]. The effects of three different *A. fumigatus* mycoviruses on the pathogenicity of as assessed using larvae of the greater wax moth *Galleria mellonella* and an uncharacterized *A. fumigatus* mycovirus shown to cause mild hypervirulent effect [44]. Since alterations were observed in both virulence and phenotype, it was important to determine whether these differences could be linked to the presence of virus derived siRNAs processed by the fungal RNA silencing machinery. The aim of this study was to characterise the sRNA populations of virus-free and virus-infected *A. fumigatus* isolates using next generation sRNA sequencing.

## Results

### sRNA libraries and quality checks

Illumina sequencing yielded a total number of reads varying between 12–30 million (M) reads. A high proportion of the reads (>99% for all samples) did not contain unassigned nucleotides; on average >80% of the reads contained the first 6 nt of the 3' adapter indicating a reliable library preparation (Table 1).

**Table 1:** Overview of the sequencing data

Sample name	Abbrev.	Lane	Total number of reads	Accepted	Proportions	Number of reads longer than 16 nt, for which the 3’ adapter was trimmed			
					Accepted	R	%R	NR	C
AfuCV-free replicate-1	CV_fr_r1	1	13,348,762	13,324,297	0.998	11,742,638	0.881	1,378,253	0.117
AfuCV-free replicate-2	CV_fr_r2	2	12,660,320	12,649,615	0.999	10,174,786	0.804	1,162,519	0.114
AfuCV-infected replicate-1	CV_inf_r1	1	18,854,816	18,820,738	0.998	16,692,438	0.886	1,542,300	0.092
AfuCV-infected replicate-2	CV_inf_r2	2	29,833,968	29,808,782	0.999	16,527,962	0.554	2,056,245	0.124
AfuTmV-1-free replicate-1	NK_fr_r1	1	23,496,360	23,451,950	0.998	18,143,950	0.773	1,934,229	0.106
AfuTmV-1-free replicate-2	NK_fr_r2	2	15,108,040	15,094,812	0.999	13,111,549	0.868	1,458,994	0.111
AfuTmV-1-infected replicate-1	NK_inf_r1	1	19,462,083	19,426,804	0.998	18,256,598	0.939	1,277,070	0.069
AfuTmV-1-infected replicate-2	NK_inf_r2	2	16,321,084	16,307,025	0.999	14,786,845	0.906	1,080,077	0.073
AfuPV-free replicate-1	PV_fr_r1	1	25,140,571	25,093,215	0.998	18,306,280	0.729	1,804,541	0.098
AfuPV-free replicate-2	PV_fr_r2	2	17,933,898	17,917,874	0.999	15,624,975	0.872	1,144,861	0.073
AfuPV-infected replicate-1	PV_inf_r1	1	43,825,751	43,744,985	0.998	41,804,128	0.955	1,748,249	0.041
AfuPV-infected replicate-2	PV_inf_r2	2	20,461,547	20,444,254	0.999	18,586,370	0.909	1,233,590	0.066

For each sample, we present the total number of reads, the number of accepted reads (reads without unassigned nucleotides, Ns), and the number reads longer than 16 nt, for which the 3’ adapter was found and trimmed. The redundant reads (R) are all accepted reads, the count of non-redundant reads (NR) is equivalent to the number of unique reads; the complexity (C) is the ratio of NR to R reads and can vary between 0 and 1. Values close to 0 indicate the presence of a small number of very abundant sequences and values close to 1 indicate the presence of many sequences, with low abundances

Positional nucleotide compositions of the sRNAs were evaluated for any technical errors arising from ligation bias or sequencing. In all tested size classes ranging from 17 nt to 25 nt, the nucleotides in each position show almost an equal probability of being present (Information content, IC close to 0) which confirmed that sequencing bias was low and there was no favoured or over-amplified sRNA sequences in the sequencing result.

**Fig. 1:**
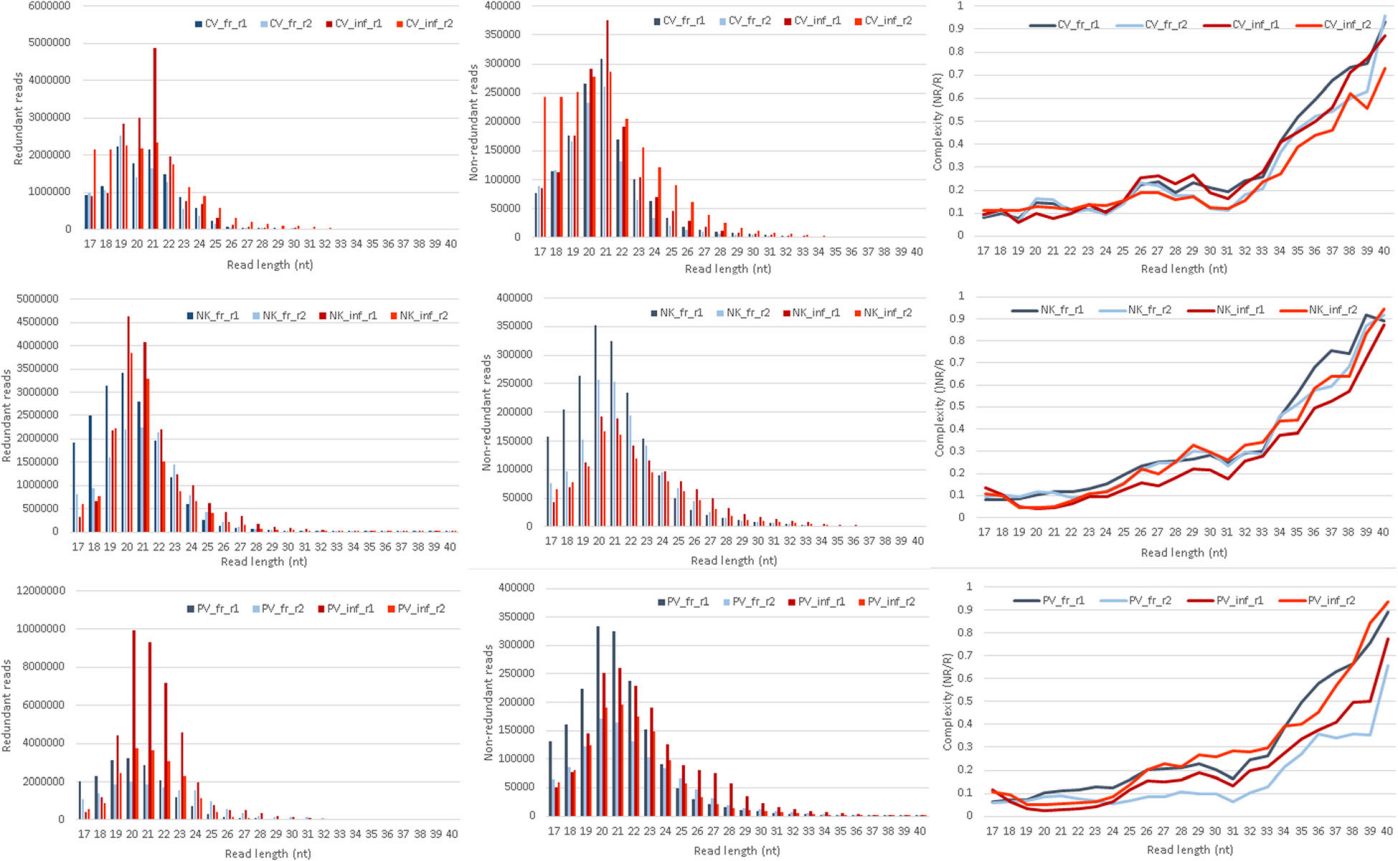
Size class (redundant, R and nonredundant, NR) and complexity (C) distributions for all samples after adapter removal and before the alignment to the reference genomes for the virus-free and virus-infected samples. The CV, NK and PV correspond to Aspergillus fumigatus chrysovirus (AfuCV), a strain of Aspergillus fumigatus tetramycovirus-1 (AfuTmV-1) and Aspergillus fumigatus partitivirus-1 (AfuPV-1), respectively. Histograms show the size class distribution of all (redundant; R) and unique (non-redundant; NR) reads and also the complexity (C) of the samples. On the *x-axis* we represent the size classes from 17 to 40 nucleotides (nt). On the *y-axis* we represent the overall abundance (for the R distribution), the frequency of reads in each size class (for the NR distribution) and the complexity (for the complexity distribution). The complexity, calculated as the ratio of non-redundant to redundant reads, varies between 0 and 1 [37]. Low complexity values (close to 0) indicate a small group of highly abundant sequences, values close to 1 indicate the presence of a large group of sequences with low abundance. (CV_fr_r1: AfuCV free isolate replicate-1; CV_fr_r2: AfuCV free isolate replicate-2; CV_inf_r1: AfuCV infected isolate replicate-1; CV_inf_r2: AfuCV infected isolate replicate-2; NK_fr_r1: AfuTmV-1 free isolate replicate-1; NK_fr_r2: AfuTmV-1 free isolate replicate-2; NK_inf_r1: AfuTmV-1 infected isolate replicate-1; NK_inf_r2: AfuTmV-1 infected isolate replicate-2; PV_fr_r1: AfuPV-1 free isolate replicate-1; PV_fr_r2: AfuPV-1 free isolate replicate-2; PV_inf_r1: AfuPV-1 infected isolate replicate-1; PV_inf_r2: AfuPV-1 infected isolate replicate-2)

**Fig. 2:**
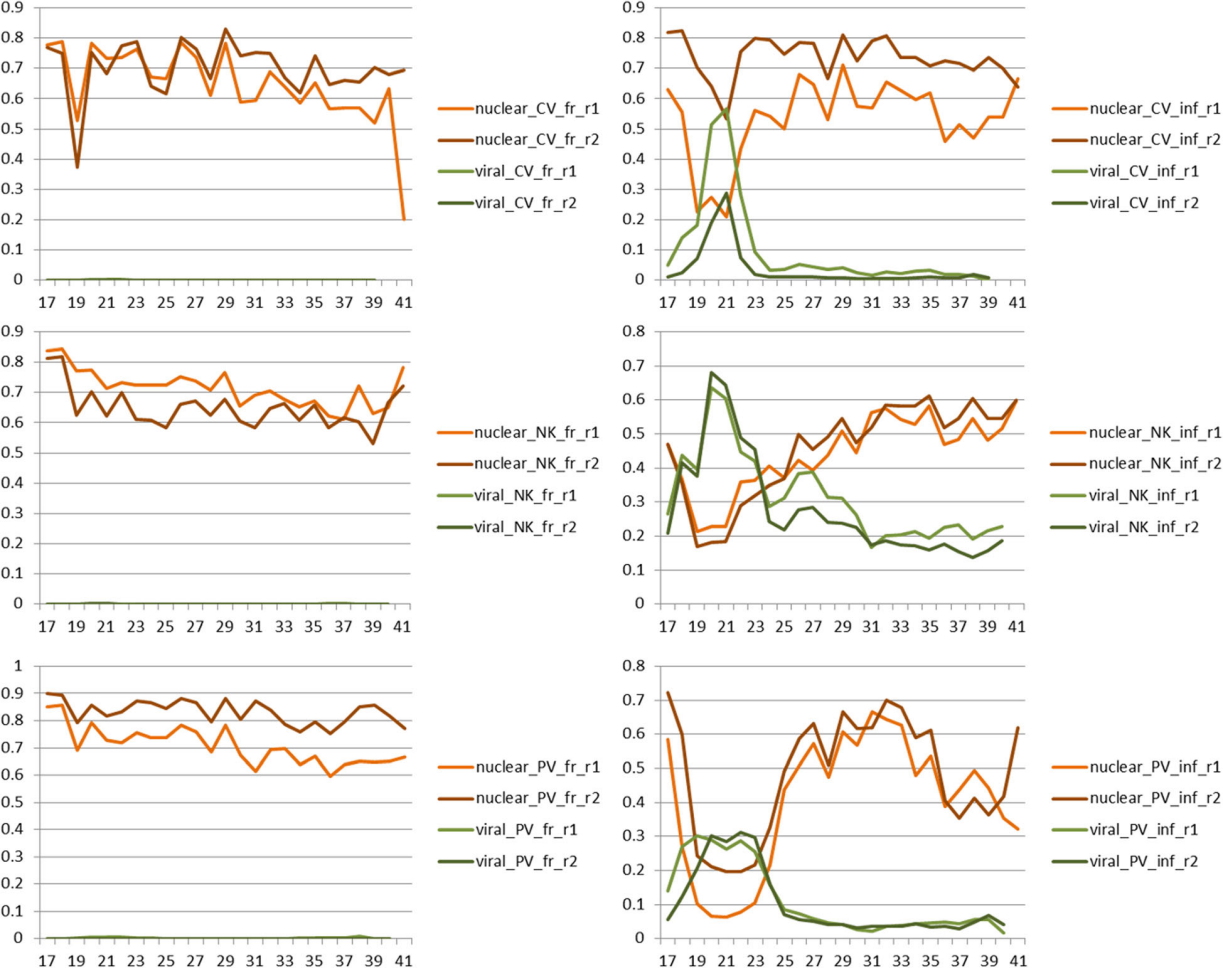
Proportions of reads matching to the reference genomes. The proportions of redundant reads matching to the nuclear genome (*y-axis*) are shown in *orange* lines; the proportions relative to the viral genomes are indicated in *green* lines. On the *x-axis* we represent the size classes. The proportions of fungal genome matching in virus-free samples were consistently high and the number of incident reads matching to the viral genomes was low (few spurious incident reads were observed). In virus-infected samples the proportion of reads matching to the viral genome peaks in the range of 19–24 nucleotide (nt) reads and corresponds to a drop in the proportions of reads matching to the fungal genome. The overall proportion of reads matched to one of the reference genomes – fungal or viral – is constantly high across all size classes. The CV, NK and PV correspond to Aspergillus fumigatus chrysovirus (AfuCV), a strain of Aspergillus fumigatus tetramycovirus-1 (AfuTmV-1) and Aspergillus fumigatus partitivirus-1 (AfuPV-1), respectively. (CV_fr_r1: AfuCV free isolate replicate-1; CV_fr_r2: AfuCV free isolate replicate-2; CV_inf_r1: AfuCV infected isolate replicate-1; CV_inf_r2: AfuCV infected isolate replicate-2; NK_fr_r1: AfuTmV-1 free isolate replicate-1; NK_fr_r2: AfuTmV-1 free isolate replicate-2; NK_inf_r1: AfuTmV-1 infected isolate replicate-1; NK_inf_r2: AfuTmV-1 infected isolate replicate-2; PV_fr_r1: AfuPV-1 free isolate replicate-1; PV_fr_r2: AfuPV-1 free isolate replicate-2; PV_inf_r1: AfuPV-1 infected isolate replicate-1; PV_inf_r2: AfuPV-1 infected isolate replicate-2)

Next, the size class distributions (before and after the matching to the reference genomes, fungal and viral) were investigated. The distributions for all (redundant) reads, before genome matching, showed that the lengths of the majority of sRNAs were between 19–23 nt. Two peaks of abundance were observed for 20mers and 21mers; these corresponded to low complexities, suggesting the existence of a small number of unique reads with high abundance (Fig. 1). Next, reads were mapped to the *A. fumigatus* nuclear genome (full length, no mismatches allowed) using PatMaN [47] with a genome matching proportion of 70% in virus-free samples. The proportion of fungal genome matching reads is in line with those reported for other fungi e.g. *Mucor circinelloides* [43] reports ~75% of reads incident to the fungal genome. The slightly lower proportion in *A. fumigatus* may also be due to the quality of the genome assembly [23]. In virus-infected samples while the proportion of reads mapping to the fungal genome decreased due to the presence of virus-derived reads the proportion of reads mapping to the corresponding viral genome increased. It was observed that sum of the proportions of genome mapping reads (both the fungal genome and viral genomes) reached 90% in all virus-infected samples and was constant across size classes (Fig. 2) indicating that the majority of reads present in the libraries were accounted for. The non-genome matching reads exhibited a higher complexity than genome matching reads and showed no enrichment for any particular size class; these characteristics indicate that these reads may be random degradation products or just sequencing noise [38].

The size class distribution of virus mapping sRNAs showed a non-random peak at 20–21 nt for AfuCV and AfuTmV-1, and at 20–23 nt for AfuPV which could be a hallmark of specific Dicer activity. This pattern was also supported by low complexity at 20–21 nt range for AfuCV and AfuTmV-1, and 20–23 nt range for AfuPV-1. Also the abundance of the reads mapping to the viral genome in virus-free samples was very low (less than 1000 reads in AfuCV and AfuTmV-1 and less than 14000 reads in AfuPV-1) whereas in virus-infected samples the abundance of the virus-matching reads were in the millions.

The size class distribution of fungus mapping sRNA population showed that the majority of the reads were in the 20–21 nt range. However low complexity was observed at 24–25 nt even though no obvious peak was observed in this size range (Additional file 1).

For normalisation, the RPM method [40] was applied first. Due to the high variability between replicates, for all size classes, this normalisation introduces many false positives for the treatment differential expression (DE) analysis. As an alternative, we employed the quantile normalisation method [14] adapted for sRNA datasets [9]. We observed that using this latter method the distribution of differential expressions for each size class was centred on 0, and replicates indicated low variability. As seen in Additional file 2 the majority of the differentially expressed reads in replicates were in the −/+ 2 log_2_ range and centred on 0 (no DE) which confirms the quantile normalisation was an appropriate normalization for the current data.

### Virus derived sRNA profiles and northern validation

Comparisons of the total reads for two biological replicates which map to the three virus genome sequences in infected samples for AfuCV, AfuTmV-1 and AfuPV-1 were respectively *ca*. 22% (3,588,921/16,610,200), 50% (8,309,887/16,521,722) and 25% (7,717,331/30,195,249) as compared to 0.001% (8,831/10,958,712), 0.0003% (5,634 /15,627,750) and 0.002% (27,497/16,965,628) for the virus-free counterpart samples.

**Fig. 3:**
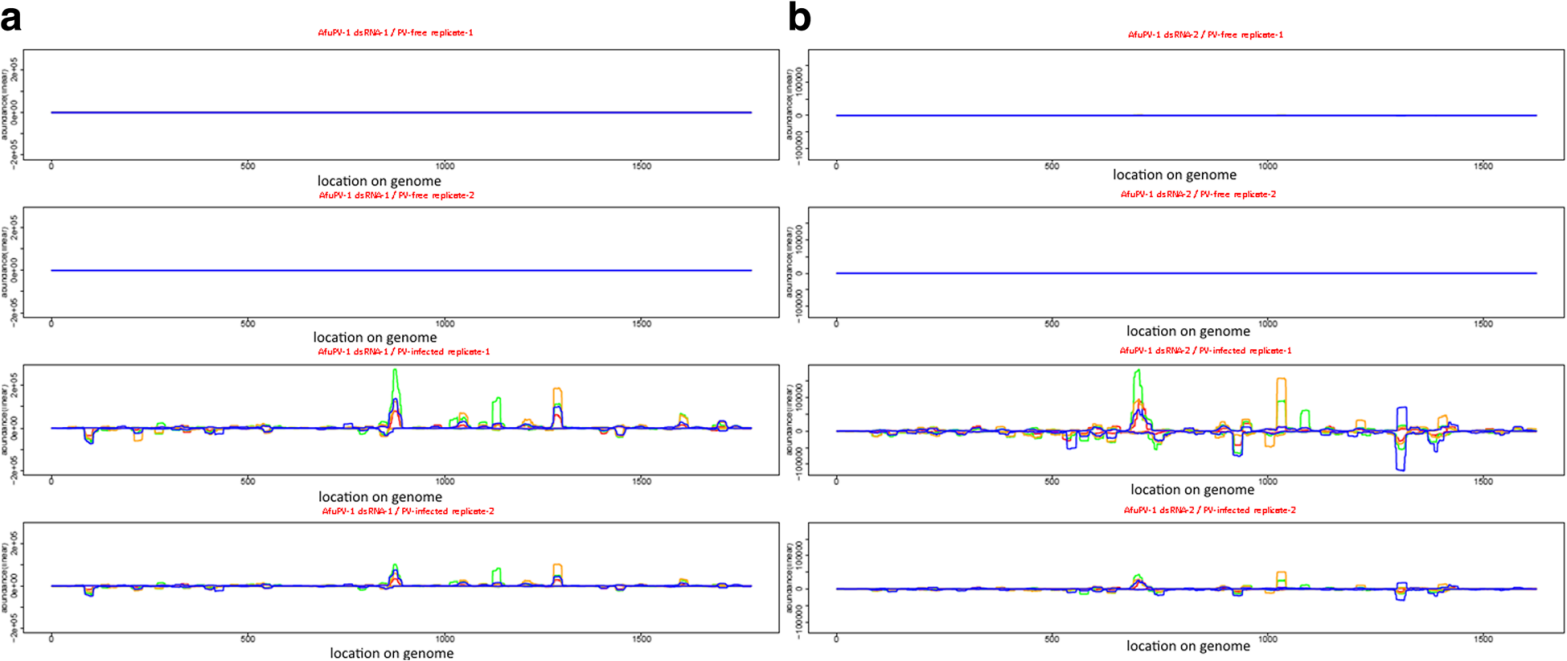
Presence plots showing the distribution and abundance of Aspergillus fumigatus partitivirus-1 (AfuPV-1)-derived sRNAs for the 2 segments of the AfuPV-1 genome. Double-stranded RNA segments 1 and 2 are presented in subplots **a** and **b**, respectively. The *red*, *green*, *orange* and *blue* lines indicate 19 nt, 20 nt, 21 nt and 22 nt siRNAs, respectively. The abundance plot of reads matching to the positive strand is presented as positive abundance, whereas the abundance plot of reads incident to the negative strand are presented as negative abundances. Genomic coordinates are shown on the *x-axis* and the point cumulative abundance of all incident reads (on a linear scale) are shown on the *y axis.*

Size-separated presence plots of PV are shown in Fig. 3. Since the other viruses have multiple dsRNA segments, the presence plots for all components are presented as Additional file 3.

For each virus, four virus-derived sRNAs were confirmed following northern hybridisation. Probes for the northern blots were designed to hybridise with the most abundant virus-derived sRNAs as identified on the presence plots (Additional file 4).

**Fig. 4:**
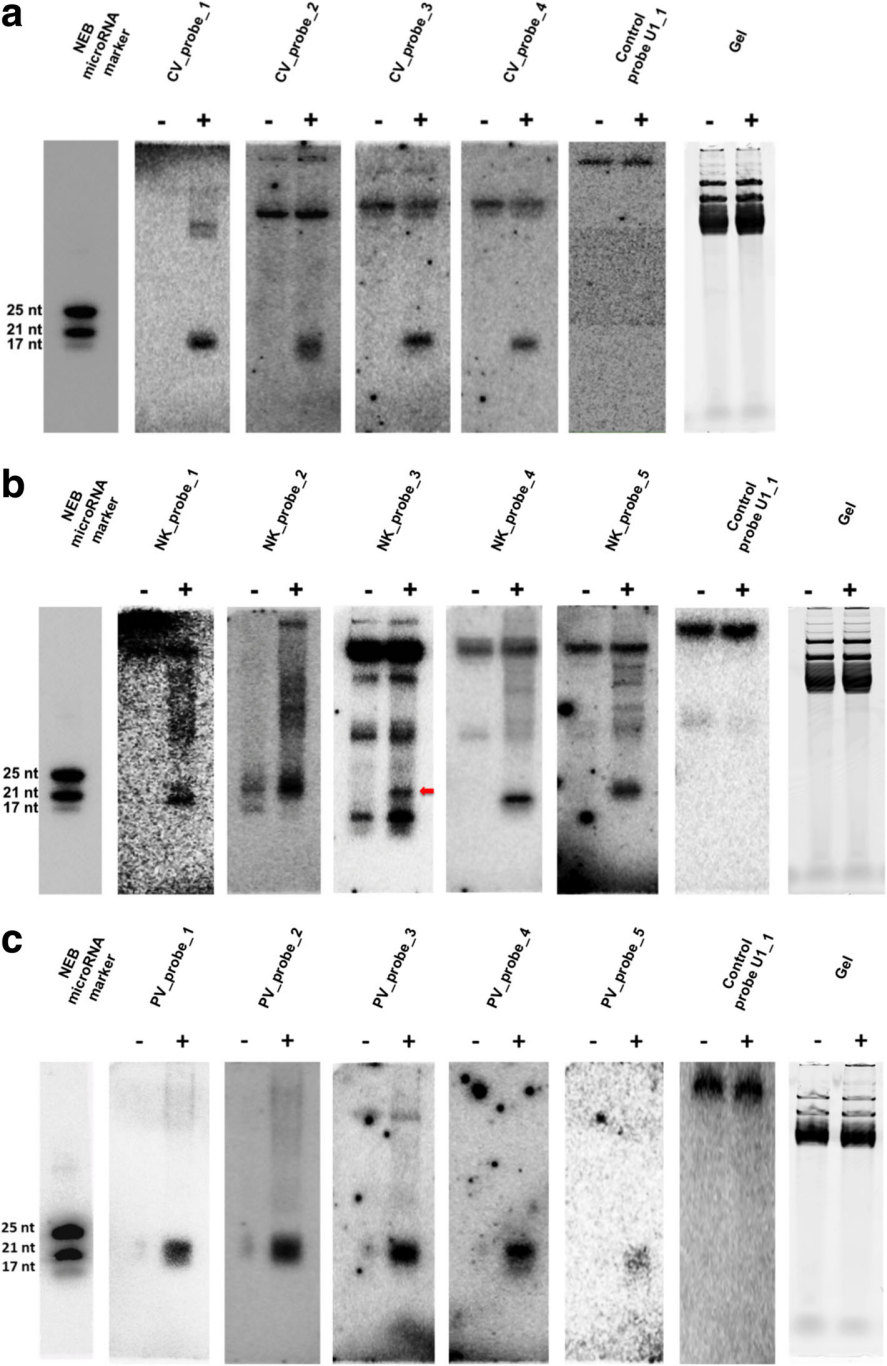
Northern blot analysis of (**a**) Aspergillus fumigatus chrysovirus (AfuCV); (**b**) Aspergillus fumigatus tetramycovirus-1 (AfuTmV-1) and (**c**) Aspergillus fumigatus partitivirus-1 (AfuPV-1) derived sRNAs. Virus-free isolates (−) and virus-infected isolates (+) are shown separately. Probes were labelled with P^32^ and the membrane was hybridised at 37 °C overnight prior to exposure to a phosphorimaging plate (GE Life Sciences) at 4 **°**C overnight using a Typhoon FLA 7000 Phosphorimager (GE Life Sciences). The same membrane was used for each probe following removal of the original probe by heating for 2 h at 90 °C

Two sRNAs identical to the negative strands of AfuPV-1 (second segment, from 176 to 194, with 0 mis-matches and 0 gaps) and AfuTmV-1 (dsRNA3, from 764 to 782, with 0 mis-matches and 0 gaps) were respectively 93.8% and 100% similar to two different regions in *A. fumigatus* long interspersed nuclear element (LINE) transposon sequences; their differential expression between the virus-free and the virus-infected samples was confirmed by northern hybridisation. The low throughput validation proved that all of the proposed siRNAs were detectable only in corresponding virus-infected samples and that the sizes were as anticipated (Fig. 4). These results were also compatible with the observed size classes on the peaks. For instance in AfuPV-1 presence plots multiple size variants were observed forming one peak. This phenomenon was visualised on northern blots of AfuPV-1 as a broader sRNA band suggestive of more than one sRNA variant (Fig. 4c; Additional file 5). Conversely, in AfuCV (Fig. 4a) and AfuTmV-1 (Fig. 4b) presence plots the peaks observed suggested the presence of one variant only and this was illustrated in northern blots as sharper bands.

### sRNAs matching to the fungal genome and microRNA-like (miRNA-like) candidates

For the identification of miRNA-like candidates, we focused our attention on sequences which were present in at least one of the sRNA-seq libraries [22, 41]. First, we checked whether any of the miRNAs described in miRbase [31] exhibited a high similarity with the sequencing reads [54], incident to either fungal or viral genomes. This analysis, conducted allowing up to 4 mis-matches and no gaps on the miRbase miRNAs, did not yield any results. We then predicted miRNA-like candidates, using the sequencing reads, and the reference genome, by applying the mirCat approach [41] with variable parameters (details described in the Methods section). This search yielded Folded-1, Folded-2 and miRCat-2 candidates (Additional file 6). The candidates Folded-1 and Folded-2 was obtained using the miRcat approach, with no restrictions, other than the length of the hairpin i.e. the only criteria which was used was the identification of a hairpin-like secondary structure. Subsequent analyses, revealed that the Folded-1 candidate exhibited high similarity to *A. fumigatus* rRNAs (a full characterization of this candidate is presented in Additional file 7).

**Fig. 5:**
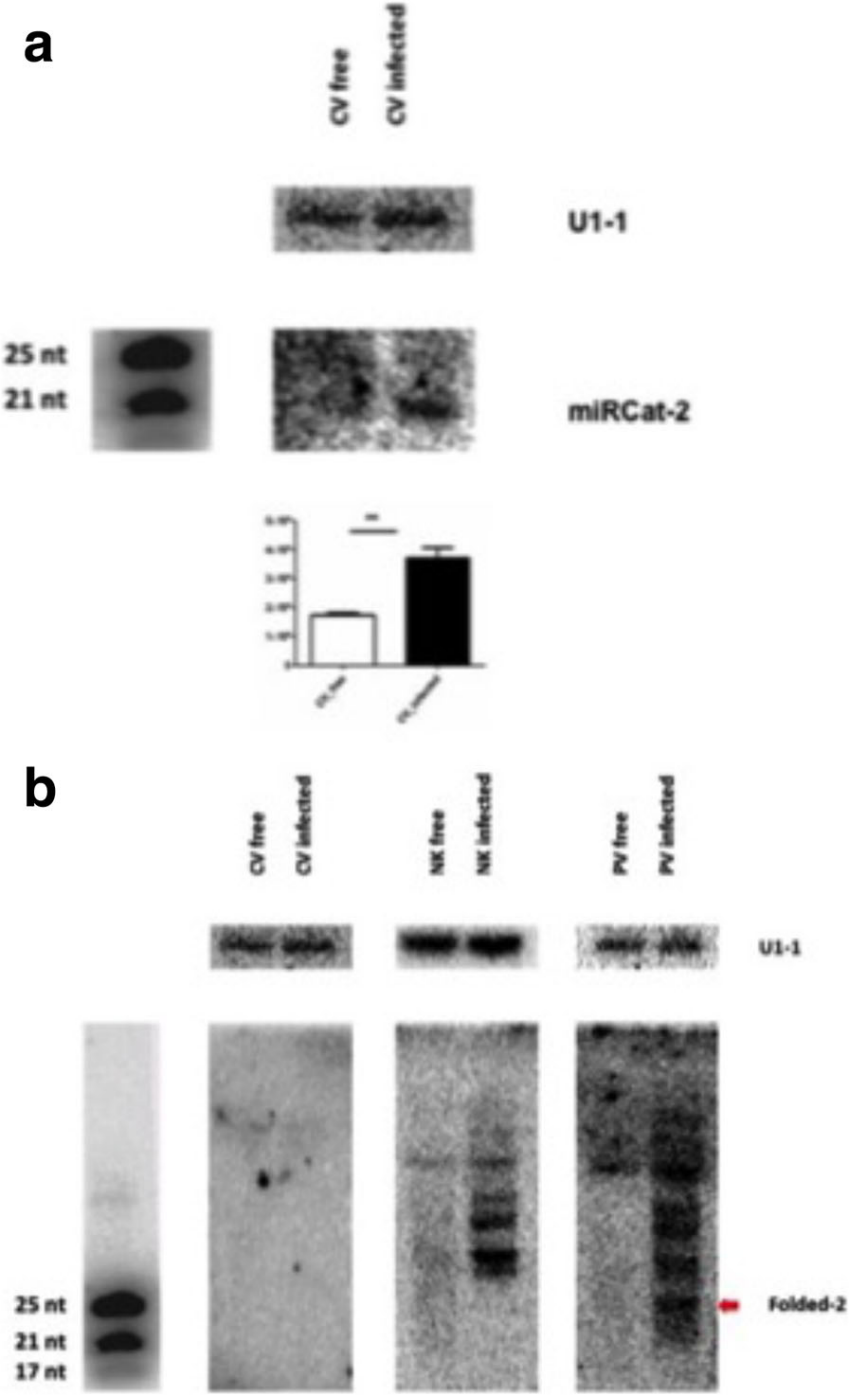
Quantitative northern blot analysis of miRNA-like candidates (**a**) miRCat-2 and (**b**) Folded-2. Individual lanes were loaded with 4 μg sRNA which were blotted and onto membranes and hybridised at 37 °C overnight with. P^32^ labelled oligonucleotides designed to detect miRNA-like candidate species. *A. fumigatus* U1-1 small nuclear RNA was used as a loading control throughout and the miRNA bands were normalised using the integrity of the U1-1 bands. Relative expression levels were plotted using the GraphPad Prism 6.0 programme and *P* values were estimated using unpaired *t*-test with Welch’s correction. Error bars were calculated using the standard error values. *P* values less than 0.05 were accepted as statistically significantly different and are shown with an asterisk (** indicates *P* ≤ 0.01; *n* = 3)

The miRNA-like candidate, miRCat-2 (20 nt long) was present in both AfuCV-infected and virus-free isolates and differentially expressed during the virus infection; the sequencing-based expression levels were confirmed by quantitative northern hybridisation (Fig. 5a).

Candidate Folded-2 was a 25 nt long miRNA-like read which is 100% identical to a region of characterised *A. fumigatus* LINE-like transposons; based on sequencing data it was predicted to be differentially expressed in all three virus-infected isolates. Northern hybridisation confirmed the presence of this miRNA-like candidate only in the AfuPV-1-infected isolate and its absence from AfuCV- and AfuTmV-1 infected isolates (Fig. 5b).

### Identification of sRNA-producing loci

The sRNA locus approach was used to identify putative regions which produce sRNAs as described previously in [38]. As there is no information available concerning the *A. fumigatus* sRNAome, including no known miRNA loci, miRNA-like or heterochromatin loci, it was relevant to determine sRNA loci on the *A. fumigatus* genome and investigate whether these are differentially expressed following virus infection. Differentially expressed loci (|DE| ≥ 2; more abundant in either the virus-free or virus-infected samples) between each virus-free and virus-infected isogenic line were annotated in order to determine the loci which may play a regulatory role in the presence or absence of virus infection (Additional file 8). Loci analysis of AfuCV infected samples revealed that sRNAs might be produced from the *sidD* gene which is involved in secondary metabolite synthesis and also the *ace1* gene which is a C2H2 transcription factor (Additional file 8A). While in AfuTmV-1 infected samples *rab7* and *gliZ* genes were determined as putative sources of sRNA, in virus-free samples regions including *cafA* and *nrps8* genes were differentially expressed. It was also discovered that in both AfuTmV-1 and AfuPV-1 infected samples LINE transposon related ORF expression was increased (Additional file 8B&C, respectively). Intersection analysis was also performed to determine any common sRNA producing regions however none were detected at the high end of DE.

## Discussion

A number of studies have demonstrated the presence of mycovirus-derived sRNAs in fungi [25, 27, 52, 64]; however none of these utilised next generation sequencing (NGS) technology to sequence sRNA libraries. To date, the accumulation of mycovirus derived sRNAs was only compared with vsiRNAs derived from plant viruses and were found to be lower in mycoviruses. Additionally these results were obtained following the analysis of a small number of siRNAs using northern hybridisation [27]. It is indisputable that NGS provides the opportunity to investigate a snapshot of the whole population of vsiRNAs viral sRNAs and is more informative than the earlier studies. Although using deep sequencing technology sRNA profiles of virus-infected plants and insects were investigated abundantly, a limited number of studies have been done in fungal systems. Recently, NGS technology has been utilised to detect fungal viruses or to sequence their genome [55], to reveal sRNA profiles in the presence of mycovirus infection [15, 20, 55, 59].

Here, sRNA profiles of three different mycovirus-infected *A. fumigatus* isolates were investigated using NGS. These viruses are all dsRNA viruses but are members of different virus families each possessing specific properties [10, 29, 30]. Therefore, it was anticipated that differences in the sRNA profiles would affect both the *A. fumigatus* host and the virus profiles. From the results it is clear that vsiRNAs sizes ranging between 19–25 nt were produced from AfuCV, AfuPV-1 and AfuTmV-1. For instance, it is notable that while sRNAs derived from AfuCV and AfuTmV-1 have a size range of 20–21 nt and gave a positive signal band following northern hybridisation analysis, sRNA derived from AfuPV-1 produces multiple size variants which can be visualised in presence plots and northern blots. It was shown previously that in *A. thaliana* DCL-1 generated 21 nt sRNAs from a ssDNA virus while DCL-4 has a role in silencing RNA viruses [13, 19]. Conversely, it has been demonstrated that in *D. melanogaster*, Dicer-2 has a role in the cleavage of diverse viruses including one DNA virus and three different RNA viruses each with different coding properties [50]. Since different size class specificities for different viruses have been demonstrated in this study it is important to investigate whether different *A. fumigatus* Dicers are responsible for these observations [24, 26]. It can be speculated that at least two different Dicers may function while one type can cleave both AfuCV and AfuTmV-1 dsRNAs and a second type might cleave AfuPV-1 dsRNA (Additional file 9).

The generation of Dicer knock-outs may be informative in understanding the differences between vsiRNAs and Dicer activities, in a similar approach as performed recently in insects [56]. This hypothesis is also supported by qPCR results of RNA silencing related genes of *A. fumigatus* which show that AfuPV-1 infection has different effects on the expression of RNA silencing genes (Additional file 9).

Another interesting point observed in sRNAome of *A. fumigatus* mycoviruses was that different viruses had distinct patterns of strand distribution despite the fact that they all have dsRNA genomes. Asymmetry in strand bias is common in silenced ssRNA plant viruses and recently it was shown that in two different insect viruses variable strand bias was also common [34, 39, 42]. It was reported that mapping of the sRNAs to *Homalodisca vitripennis reovirus* (HoVRV) genome were predominantly to the negative-strand despite the fact that it possessed a dsRNA genome [42]. Moreover, unequal strand bias was shown in mycoviruses infecting *Botrytis* genus (Donaire & Ayllon, 2016). Upon further investigations, this information could be linked to the replication properties of dsRNA viruses as it may be the indicator that encapsidated dsRNA would be exposed to the cytoplasm in another form such as a replication intermediate which could be targeted by host RNA silencing.

One hypothesis emerging from this study is that vsiRNAs may influence host mRNA expression and lead to phenotypic changes in *A. fumigatus.* Such alterations following virus-infection may be the result of simultaneous silencing of mycovirus dsRNAs and sequence-specific degradation of host mRNAs. Initial checks, conducted using qPCR, revealed that the expression levels of some genes potentially targeted by virus-derived sRNAs were reduced following virus infection (Additional file 9). For instance *pskP*, a key gene involved in pigmentation and also pathogenesis is down-regulated in AfuCV infected fungal isolates (Additional file 9). However, this down-regulation was only marginally significant (*P value* = 0.17, under two-tailed Student’s *t*-test with unequal variance). Another gene assayed using qPCR assays was the *pyrG* gene, important for filamentous growth and pathogenicity (Additional file 9). Its expression level for Aspergillus fumigatus partitivirus-1 infection was reduced significantly (*P value* = 0.000046, under two-tailed Student’s *t*-test with unequal variance).

The analysis of sRNA loci allowed us to identify putative sRNA producing regions [38] in the host genome during virus infection. A notable observation from these analyses was the potential link between the viruses and LINEs through the identification of sRNAs with high similarity to both. These sRNAs were induced by the virus infection and their differential expression between infected and non-infected samples was confirmed. Due to the high similarity to both the *A. fumigatus* genome and the viruses, it is hard to distinguish which location was the source and/or potential target. The second important observation was that pathogenicity and secondary metabolite process-related genes were incident with sRNA loci on the *A. fumigatus* genome. Although, for sRNA loci predictions, the source and target cannot be unequivocally distinguished using sequencing data, it is accepted that a region predicted based on perfect alignments of the incident reads (full length, no mis-matches) has a high probability of being the source locus [38, 43]). In fungi, secondary metabolites were shown to be advantageous in extreme conditions and also essential for fungal pathogenicity. Secondary metabolites such as melanin, which contributes to fungal pathogenicity, siderophores involving in iron metabolism or gliotoxin enhancing virulence were shown to be important contributors of pathogenicity in *A. fumigatus* [51]. In addition, it has been reported that, under normal growth conditions, secondary metabolite biosynthesis genes which cluster in the fungal genome are silent and not transcribed [16]. The sRNA loci predicted in this study may be activated during virus infection, as a stress response.

## Conclusions

Here we report for the first time virus-derived sRNA in the presence of AfuCV, AfuPV-1 and AfuTmV-1 and also first miRNA-like candidates in *A. fumigatus*. In addition, putative sRNA loci in the host genome during virus infection were identified. As differences in phenotype such as pigmentation and growth rate were noted between virus-free and virus-infected isogenic lines, it was considered that *A. fumigatus* dsRNA mycoviruses can affect gene expression *via* RNA silencing and can also affect pathogenicity. The work presented here inputs a wide range of information on sRNA profiles of *A. fumigatus* mycoviruses while offering preliminary data about milRNAs and possible targets of the sRNAs. Taken together, our data provide insights into RNA silencing against mycoviruses in an important human pathogenic fungus *A. fumigatus*.

## Methods

### *Aspergillus fumigatus* strains and growth conditions

All *A. fumigatus* strains (Table 2) were spread inoculated onto 25 ml of solid Aspergillus complete medium (ACM) using 20 μl of inoculum from 50% glycerol stocks. Plates were incubated at 37 °C for 3–4 days and spores were harvested with a glass spreader into 15 ml of sterile distilled water. Spore suspensions were used to inoculate 500 ml of ACM broth in bottles which were incubated at 37 °C with shaking at 130 rpm. After 5 days, mycelia were harvested using sterile Miracloth (Merck), rapidly frozen in liquid N_2_ and kept at −80 °C until processing. Presence and absence of the viruses were checked prior to experiments by using multiplex RT-PCR (Özkan & Coutts, unpublished).

**Table 2:** *Aspergillus fumigatus* strains investigated in this study

Nominated name	Abbreviation	Fungal strain	Genotype	Reference
AfuCV free	CV - free	A56-C	Aspergillus fumigatus chrysovirus (AfuCV) free isolate	[29]
AfuCV infected	CV - infected	A56	Aspergillus fumigatus chrysovirus (AfuCV) infected isolate	[29]
AfuTmV-1 free	NK - free	NK125	Aspergillus fumigatus tetramycovirus-1 (AfuTmV-1) free isolate	[30]
AfuTmV-1 infected	NK - infected	Af293 (Wild type)	Aspergillus fumigatus tetramycovirus-1 (AfuTmV-1) infected isolate	[30]
AfuPV-1 free	PV - free	Af237y	Aspergillus fumigatus partitivirus-1 (AfuPV-1) free isolate	[6]
AfuPV-1 infected	PV - infected	Af237y-88	Aspergillus fumigatus partitivirus-1 (AfuPV-1) infected isolate	[11]

### RNA extraction and small RNA library construction

Total RNA was extracted from 100 mg of mycelium using Trizol according to the manufacturer’s protocol. From the total RNA preparations sRNAs were enriched using the PEG-8000 precipitation method with slight modifications [36]. Briefly, high molecular weight RNAs were precipitated by adding 50% PEG-8000 to a final concentration of 10% and 5 M NaCl to a final concentration of 0.5 M. Low molecular weight-RNAs in the supernatant were then precipitated with 2.5 volumes of 100% ethanol. The integrity of total RNAs was checked on agarose gels to confirm that the samples were not degraded and the concentrations and purity were measured using a NanoDrop 2000C spectrophotometer (Thermo Fischer) before library construction.

The sRNA libraries from 2 μg sRNAs were generated using the ScriptMiner Small RNA-Seq Library preparation kit (Epicentre Biotechnologies, USA) according to the manufacturer’s protocol. The 5’ adapter was 5’GUUCAGAGUUCUACAGUCCGACGAUC3’ and the 3’ adapter was 5’AGATCGGAAGAGCACACGTCT3’. The cDNA libraries were amplified using ScriptMiner index primers including different barcodes to tag different samples. PCR amplification was performed using Phusion High-Fidelity DNA polymerase (NEB) and different cycles were set to determine the optimal cycle number of each sample. At least 2 PCR amplification reactions per sample were performed in order to maximise library yields. Following PCR amplification, libraries were size fractioned by electrophoresing the products twice through 10% native polyacrylamide gels and excising the bands of interest. Sequencing was done by the Earlham Institute (EI, Norwich) previously known as The Genome Analysis Center (TGAC, Norwich) using Illumina HiSeq2500 sequencing.

### Bioinformatics analysis

The sRNA sequencing output files were delivered in FASTQ format. The sequencing was directional, single ended and the length of the reads was 50 nt. The data was analysed on a Linux server, using Perl (version Strawberry Perl 5.18.2.1) and R (version 3.0.3) custom made scripts. The UEA sRNA Workbench was used for the prediction of sRNA loci [38, 54].

Sets of 6 samples, corresponding to each biological replicate, were loaded on a sequencing lane. To evaluate the equal loading of each library in each lane, we compared the distribution of total number of reads per library to a random uniform distribution (on the same lane sequencing depth), using the *χ* ^2^ test.

To process the reads, the adapters were trimmed using the first 6 nt of the 3’ adapter sequence. Next, the reads were mapped (full length) to the fungal genome and three different viral genomes using PatMaN [47] allowing 0 gaps and 0 mismatches for the fungus and 0 gaps and 0, 1 or 2 mismatches for the viruses. The genomes used as references for mapping were: (i) *A. fumigatus*, (ii) AfuCV, (iii) AfuPV-1 and (iv) AfuTmV-1. The *A. fumigatus* genome (Af293, version s03-m04-r22) has a length of 29.4 Mb and is divided into 8 chromosomes together with a 32 kb mitochondrial genome [23]. The full genome and corresponding annotations were downloaded from the *Aspergillus* Genome Database (AspGD; [5]). The sequences of the AfuCV, AfuPV-1 and AfuTmV-1 NK viruses were obtained from the NCBI database using the accession numbers cited in [10, 29] and [30], respectively. Next, the positional nucleotide composition separated per size class, were analysed. The quality of the resulting sequences was checked before and after genome matching by evaluating the size class distributions on the redundant (all) reads, non-redundant (unique) reads, as well as the distribution of complexities [37].

The next step consisted of a comparison of the replicates followed by the identification of differential expression (DE) between the treatments - virus-free versus virus-infected samples. A first measure to assess the replicate versus treatment sample similarity and localise the differences between the samples was the Jaccard similarity index which was calculated on the top 1000 and 5000 most abundant reads [37]. Next, the replicate versus replicate scatter plots were created and the overall and localised Pearson correlations coefficients were computed. Normalisation was conducted using both per total normalisation [40] on an *a priori* fixed total calculated as the mean of sample sequencing depths and quantile normalisation [14] adapted for sRNA datasets. The distributions of differential expression between replicates (calculated as an offset fold change, offset = 20 [37] for each size class were centred on 0, in accordance with the hypothesis that the majority of reads (sRNAs) should not be differentially expressed between biological replicates. The quantile normalisation method was preferred due to the proximity of the median and IQR (inter-quartile range) to the 0 non-DE line of the DE distributions. Subsequent to the normalisation step, the size class distributions were recalculated and the distributions of abundances post-normalisation were compared to the corresponding pre-normalisation distributions and no significant differences were observed. DE sRNAs between the virus-free and virus-infected samples were identified using two different methods: (i) “offset fold change method (OFC)”, with an offset of 20, calculated for all replicate-permutations for virus-free and virus-infected samples [37], (ii) differential expression using confidence intervals (CI), for which the DE was computed on the proximal ends of the maximal CIs created on replicates [35]. The sRNAs were accepted as DE if they were identified by both methods. A subset of DE reads was selected for further validation.

miRNA identification was performed using three approaches; (i) similarity search against the miRBase database and known fungal miRNA-like entries, using a maximum of 3 mis-matches and no gaps (ii) miRNA prediction using miRCat [41], (iii) additional analysis of expressed sRNAs, exclusion of those matching to existing annotations, and a search for a hairpin-like secondary structure by folding of the left and right flanking regions of the remaining reads. All loci which folded into a secondary structure with an adjusted minimum free energy (aMFE) less than −20 were visually evaluated.

To detect conserved miRNAs, reads incident to either genome (fungal or viral) were mapped to all mature miRNA sequences in the miRBase database ([31]; release 20) allowing up to 4 mismatches and no gaps. The putative miRNAs were then mapped back to the reference genomes. A single mismatch was allowed because no miRNAs have been reported in fungi apart from microRNA-like (milRNAs; [33, 61, 66]). No putative miRNAs were found to be incident to the viral genomes. For the host genome, potential precursors were determined starting with left/right flanking regions of 300 nt, incrementally reducing them up to a minimum of 50 nt; the secondary structure was determined using the Vienna RNA folding suite (version 2.0; [28]). Identification of putative novel miRNAs was performed with miRCat (miRNA categoriser; [41]) using the parameters such as length of hairpin and GC content determined from the putative conserved miRNAs.

To identify potential siRNAs with a regulatory role at transcriptional level, we analysed the size class and complexity distributions of reads mapping to transposable elements, repeat elements and putative promoter regions defined as 2 kb upstream of the start codon, on the same strand as the gene. For interesting candidates, size separated presence plots were created.

To identify potential new classes of sRNAs, genome-matching reads were used for the prediction of sRNA loci using the locus identification tool (CoLIde) described in Mohorianu et al. [38]. Differentially expressed loci were annotated based on sequence similarity using BLAST, components BLASTN and BLASTX against the nr and nt databases (release 2.2.29; [4]). The cut offs used were: 80% identity, 80% similarity and a length of the homologous region of more than 18 nt.

Additional analyses were done to identify any putative new classes of vsiRNAs. With this aim, the viral presence plots were created. Next, the most abundant sequences mapping to the viral genomes were checked for differential expression using the normalized expression levels obtained from the analysis described in Section 2.6.2. Validation was done by northern blot analysis and potential targets were searched on the host genome using both plant and animal targeting rules [3, 8].

### Northern blot analysis

Northern blotting was used to validate the presence of vsiRNAs. Briefly, 2 μg of sRNA from virus-free and virus-infected *A. fumigatus* isolates were fractionated by electrophoresis through 7 M urea 16% denaturing polyacrylamide gels. Following electrophoresis the RNAs were transferred onto Hybond NX membrane (Amersham) using the semi-dry transfer system (Bio-Rad). Cross-linking was performed using 1-ethyl-3-(dimethylaminopropyl) carbodiimide at 60 °C for 2 h as described previously [45]. Hybridisation was done at 37 °C overnight using P^32^ labelled probes which were reverse complement oligonucleotides to the sRNAs of interest. To assess the relative expression levels, quantifications were done three times using ImageQuant software (version 8.1, GE Healthcare Life Sciences) and averages were plotted. The sequences of the probes used and validated sRNAs are listed in Additional file 4.

### Statistical analysis

GraphPad Prism 6.0 software was used to analyse the qPCR results and *P* values were estimated using unpaired *t*-test with Welch’s correction. Error bars were calculated using the standard error values. *P* values less than 0.05 were accepted as statistically significantly different and are indicated with an asterisk.

## Additional files

Additional file 1: Size class distribution (redundant, R and nonredundant, NR) and complexity (C) of the samples before and after genome matching to the fungal and viral genomes, respectively. Biological replicates were represented as two shades of same colour. Blue: all reads, green: reads matching to the fungal nuclear genome, red: reads matching to viral genomes. The CV, NK and PV correspond to Aspergillus fumigatus chrysovirus (AfuCV), a strain of Aspergillus fumigatus tetramycovirus-1 (AfuTmV-1) and Aspergillus fumigatus partitivirus-1 (AfuPV-1), respectively. (PDF 632 kb)

Additional file 2: Quantile normalisation. MA plots and distributions of DE (calculated using offset fold change method, OFC) separated per size class. In the MA plots, the average abundance (in log 2) is shown on the axis and the OFC (with offset = 20) is shown on the y axis. The CV, NK and PV correspond to Aspergillus fumigatus chrysovirus (AfuCV), a strain of Aspergillus fumigatus tetramycovirus-1 (AfuTmV-1) and Aspergillus fumigatus partitivirus-1 (AfuPV-1), respectively. (PDF 5547 kb)

Additional file 3 Presence plots indicating the distribution and abundance of (A) Aspergillus fumigatus chrysovirus (AfuCV) and (B) Aspergillus fumigatus tetramycovirus-1 (AfuTmV-1)-derived sRNAs along the 4 segments of the related genomes. Double-stranded RNA segments are shown as 1, 2, 3 and 4 respectively. Colour code for the lines are red, green, orange and blue for 19 nt, 20 nt, 21 nt and 22 nt, respectively. Genomic coordinates were represented on the x axis. On the y axis, the point cumulative abundance of all incident reads (in linear scale) was represented. (PDF 8465 kb)

Additional file 4: List of probes designed to validate selected virus-derived sRNAs. The CV, NK and PV correspond to Aspergillus fumigatus chrysovirus (AfuCV), a strain of Aspergillus fumigatus tetramycovirus-1 (AfuTmV-1) and Aspergillus fumigatus partitivirus-1 (AfuPV-1), respectively. (PDF 37 kb)

Additional file 5: List of most abundant variants (with abundance greater than 100) which can hybridize with the PV probes. PV correspond to Aspergillus fumigatus partitivirus-1 (AfuPV-1). For each PV probe we present shorter and longer variant sequences, with up to 2 mis-matches relative to the probe. For each sequence we show the normalized abundance in the two infected replicates and the variant length. (PDF 38 kb)

Additional file 6 Characteristics of miRNA-like candidates in *A. fumigatus*. For each miRNA-like candidate we present the hairpin sequence, the mature sequence, incident annotation based on version Af293, version s03-m04-r22 of the *A. fumigatus* genome, the samples for which the candidate is differentially expressed and the corresponding secondary structure. (PDF 217 kb)

Additional file 7: Characteristics of Folded-1 (PDF 787 kb)

Additional file 8: Histograms showing the differentially expressed (−2 ≥ DE or DE ≥ 2) sRNA loci and annotations. The sRNA loci of all three viruses were identified and expressions plotted for CV (A), NK (B) and PV (C). The CV, NK and PV correspond to Aspergillus fumigatus chrysovirus (AfuCV), a strain of Aspergillus fumigatus tetramycovirus-1 (AfuTmV-1) and Aspergillus fumigatus partitivirus-1 (AfuPV-1), respectively. Annotations of the regions of putative sources of sRNAs were presented in red to indicate those differentially expressed in virus-infected cases and in blue to indicate the ones differentially expressed in virus-free examples. Comparisons were made for all three combinations (1_1: virus_free_rep1 and virus_infected_rep_1; 1_2: virus_free_rep1 and virus_infected_rep_2; 2_1: virus_free_rep2 and virus_infected_rep_1; 2_2: virus_free_rep2 and virus_infected_rep_2). (PDF 1446 kb)

Additional file 9: qPCR validations of RNAi genes and genes responding to the virus infection. (PDF 501 kb)

## Abbreviations

ACM: Aspergillus complete medium
AfuCV (CV): Aspergillus fumigatus chrysovirus
AfuPV-1 (PV): Aspergillus fumigatus partitivirus-1
AfuTmV-1 (NK): Aspergillus fumigatus tetramycovirus-1
C: Complexity
cDNA: Complementary deoxyribonucleic acid
CI: Confidence intervals
DE: Differential expression
dsRNA: Double-stranded ribonucleic acid
IC: Information content
kb: kilobyte
LINE: Long interspersed nuclear element
miRNA: Micro ribonucleic acid
NGS: Next generation sequencing
NR: Nonredundant
nt: Nucleotide
OFC: Offset fold change
ORF: Open reading frame
qPCR: Quantitative polymerase chain reaction
R: Redundant
RdRP: Ribonucleic acid-dependent ribonucleic acid polymerase
RNA: Ribonucleic acid
RNAi: Ribonucleic acid interference
RPM: Reads per million
RT-PCR: Reverse transcription polymerase chain reaction
siRNA: Small interfering ribonucleic acid
sRNA: Small ribonucleic acid
sRNA-seq: Small ribonucleic acid sequencing
ssRNA: Single stranded ribonucleic acid
vsiRNA: Virus-derived small ribonucleic acid
