# Supplementary figures and images for: Profile and functional analysis of small RNAs derived from Aspergillus fumigatus infected with double-stranded RNA mycoviruses
Source: BMC Genomics. 2017 May 30;18:416. doi: 10.1186/s12864-017-3773-8 (PMC5450132; doi:10.1186/s12864-017-3773-8)

## CV\_free

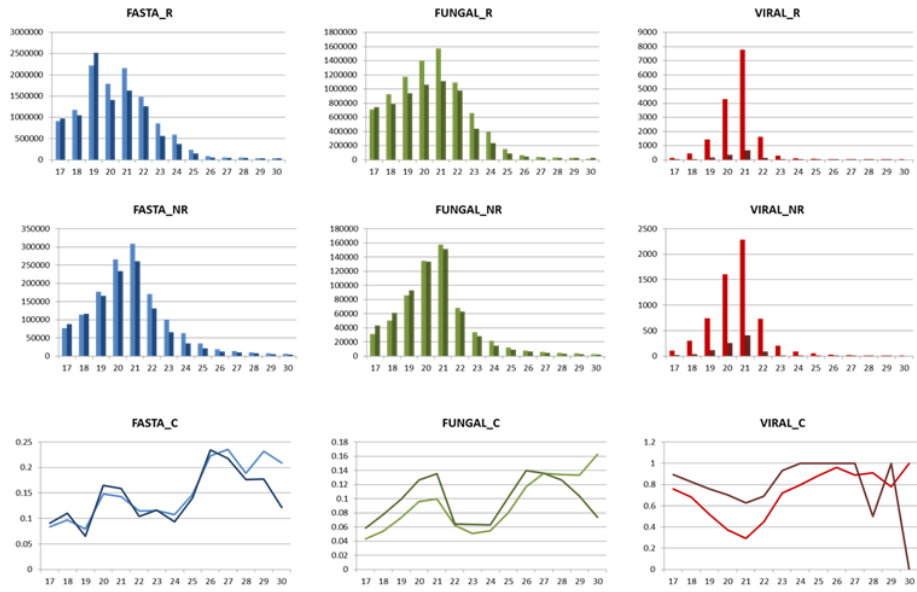

## CV\_infected

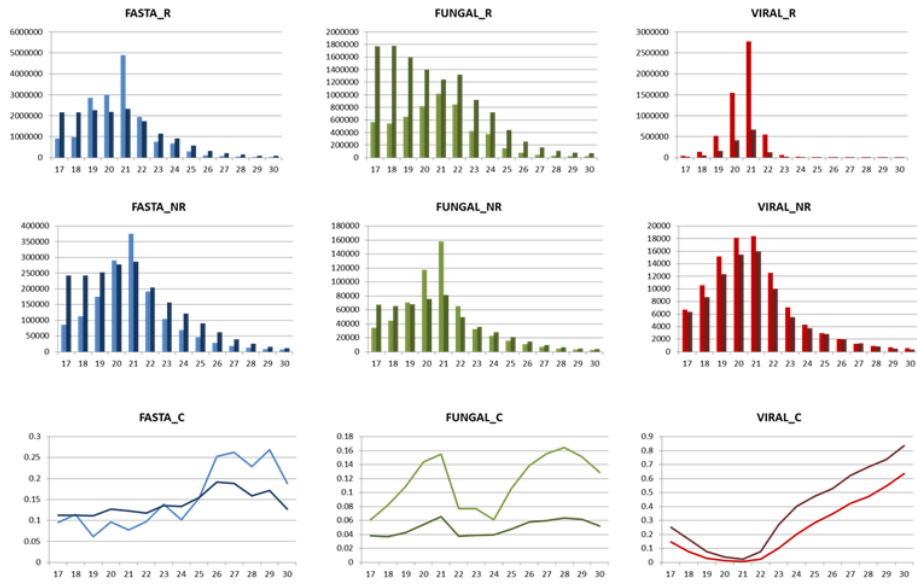

## NK\_free

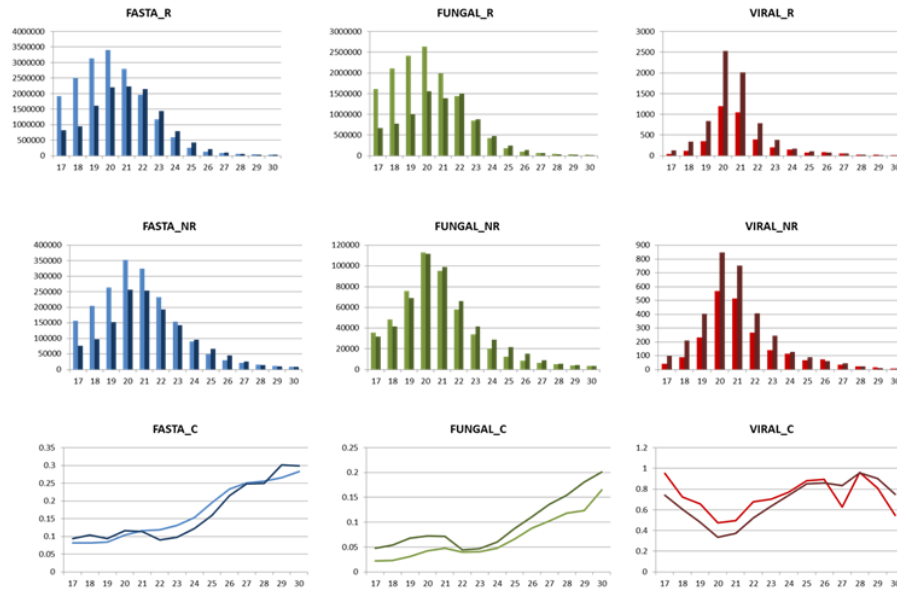

## NK\_infected

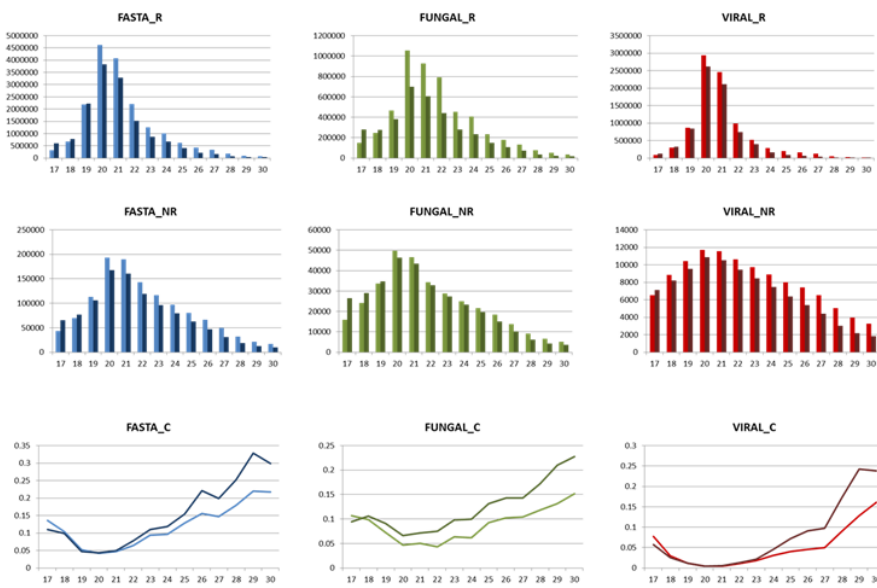

## PV\_free

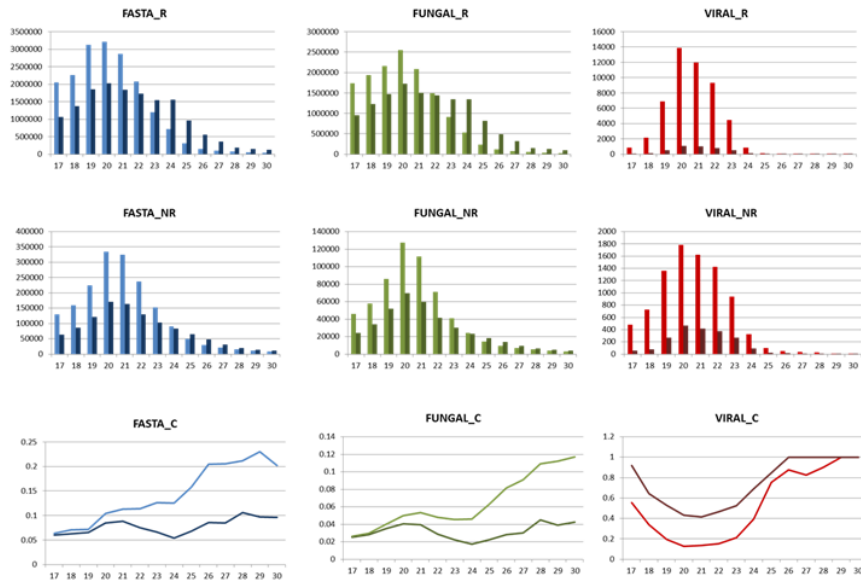

## PV\_infected

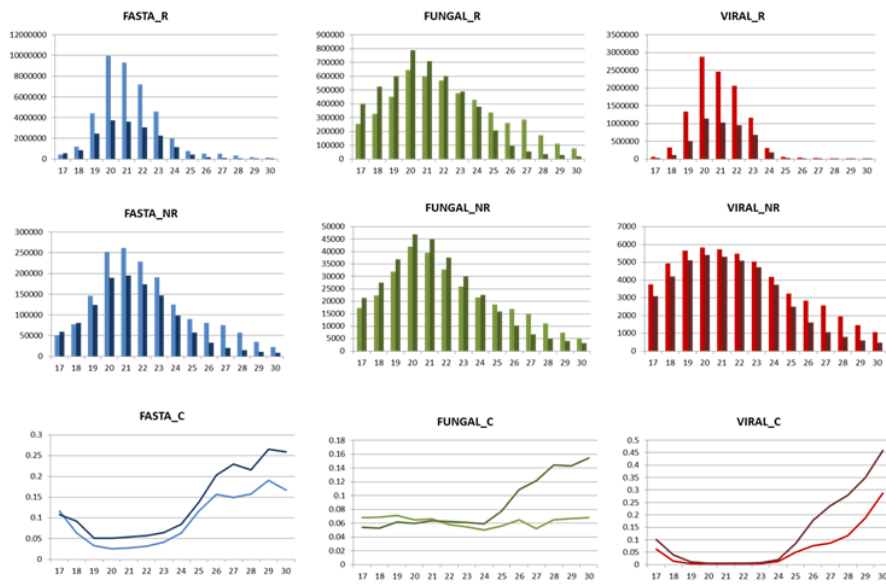

Supplement: Supplementary file 1 — Size class distribution (redundant, R and nonredundant, NR) and complexity (C) of the samples before and after genome matching to the fungal and viral genomes, respectively. Biological replicates were represented as two shades of same colour. Blue: all reads, green: reads matching to the fungal nuclear genome, red: reads matching to viral genomes. The CV, NK and PV correspond to Aspergillus fumigatus chrysovirus (AfuCV), a strain of Aspergillus fumigatus tetramycovirus-1 (AfuTmV-1) and Aspergillus fumigatus partitivirus-1 (AfuPV-1), respectively. (PDF 632 kb) [file 12864_2017_3773_MOESM1_ESM.pdf]

CV free

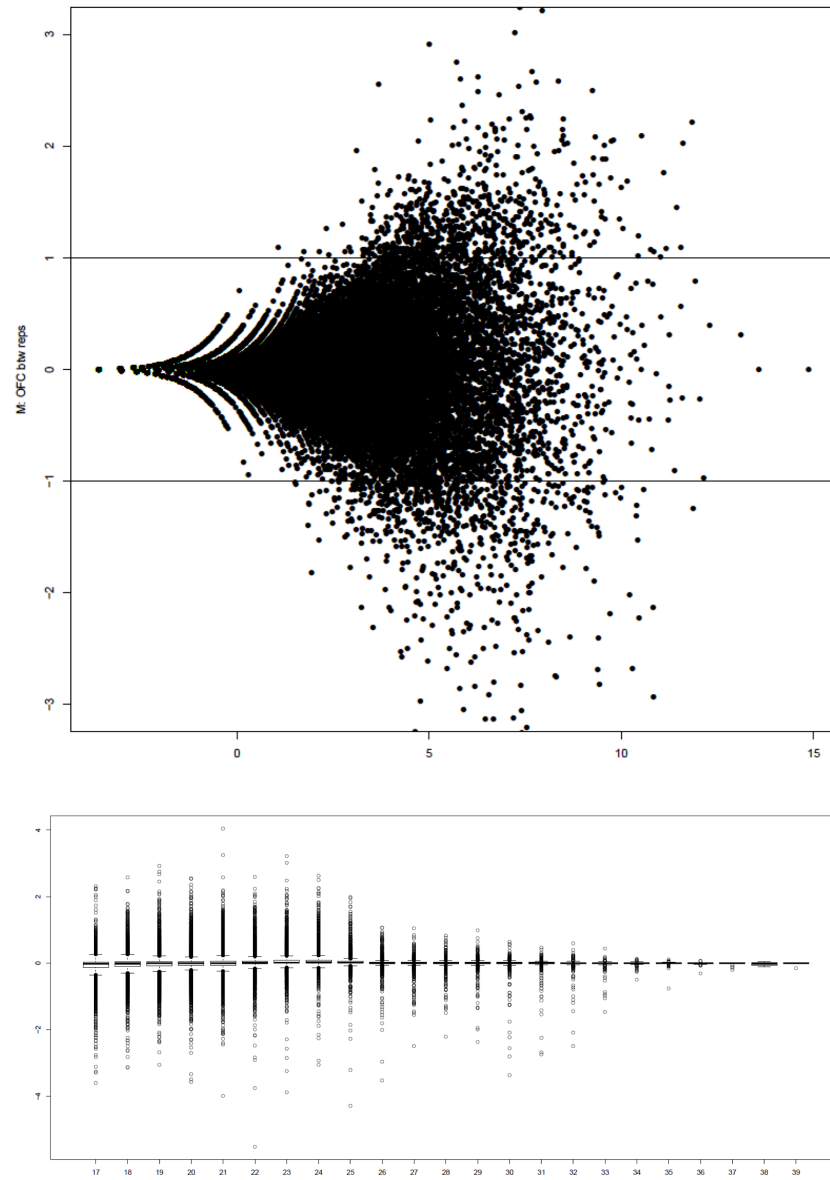

CV infected

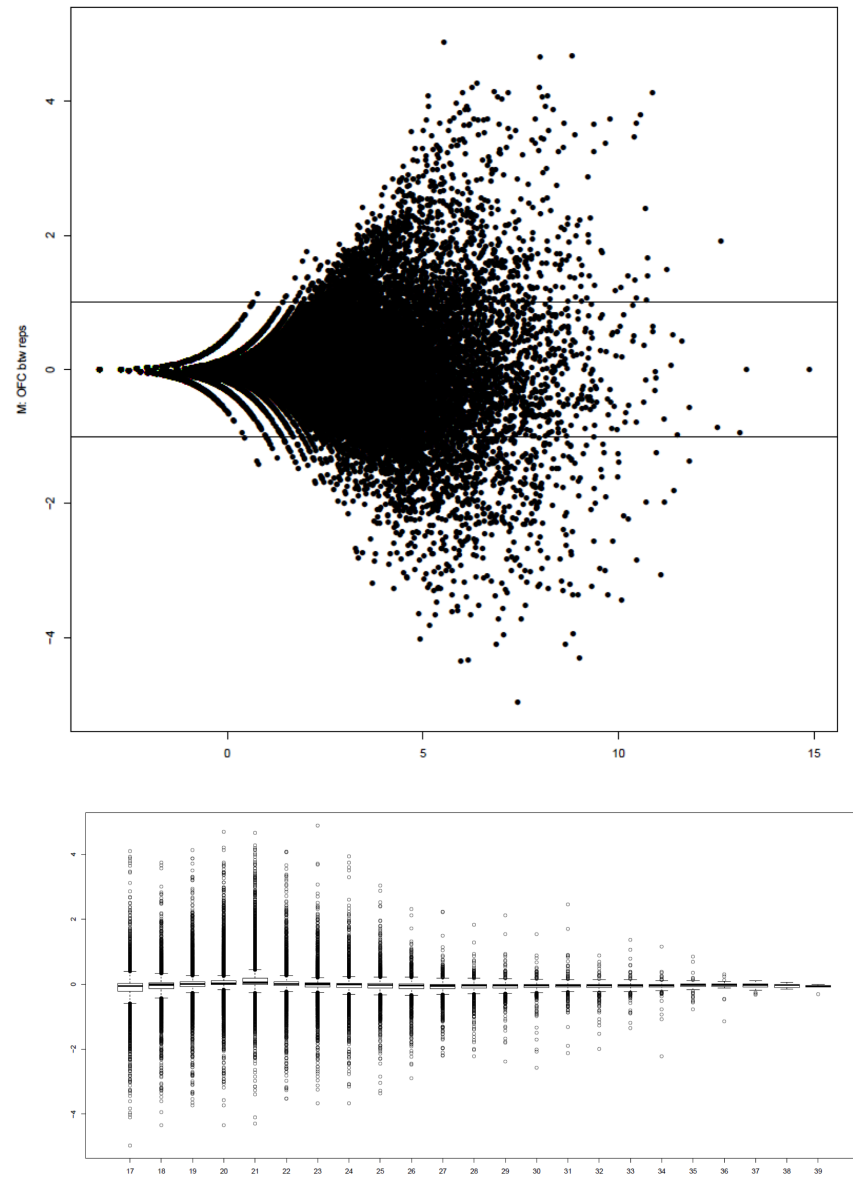

# NK free

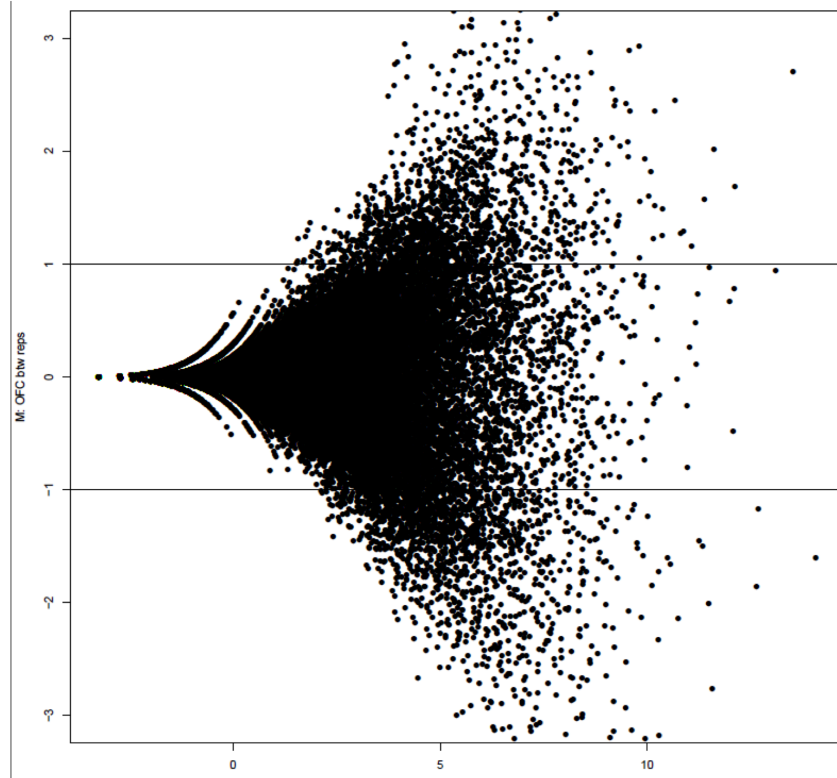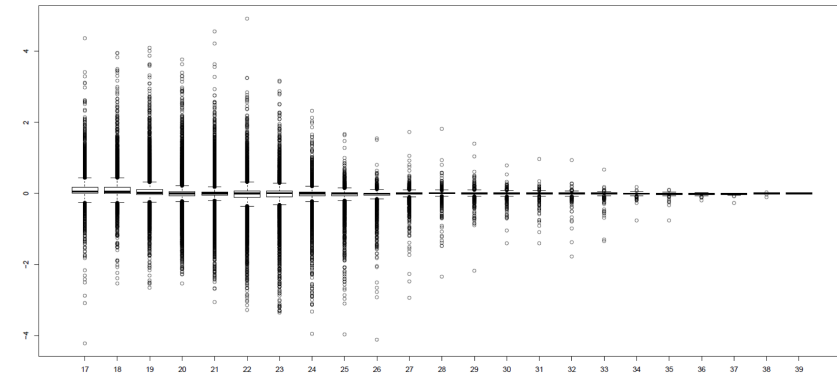

# NK infected

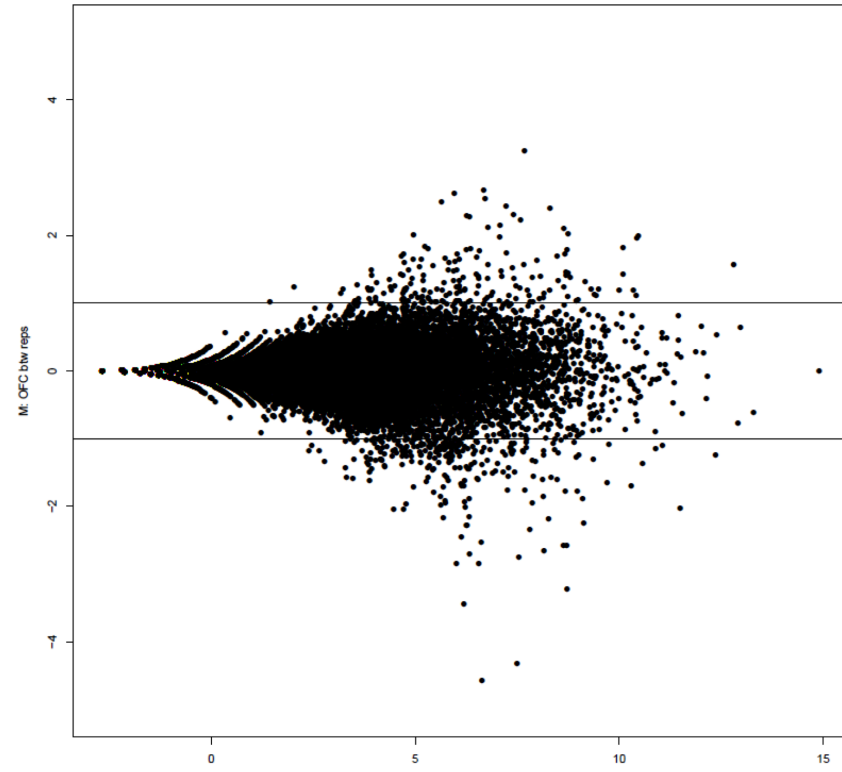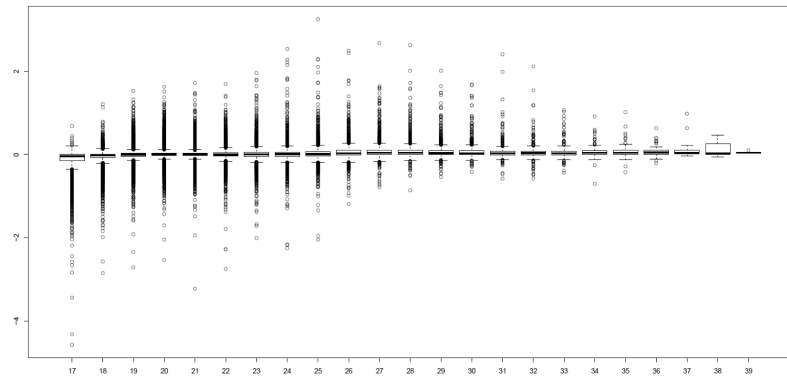

PV free

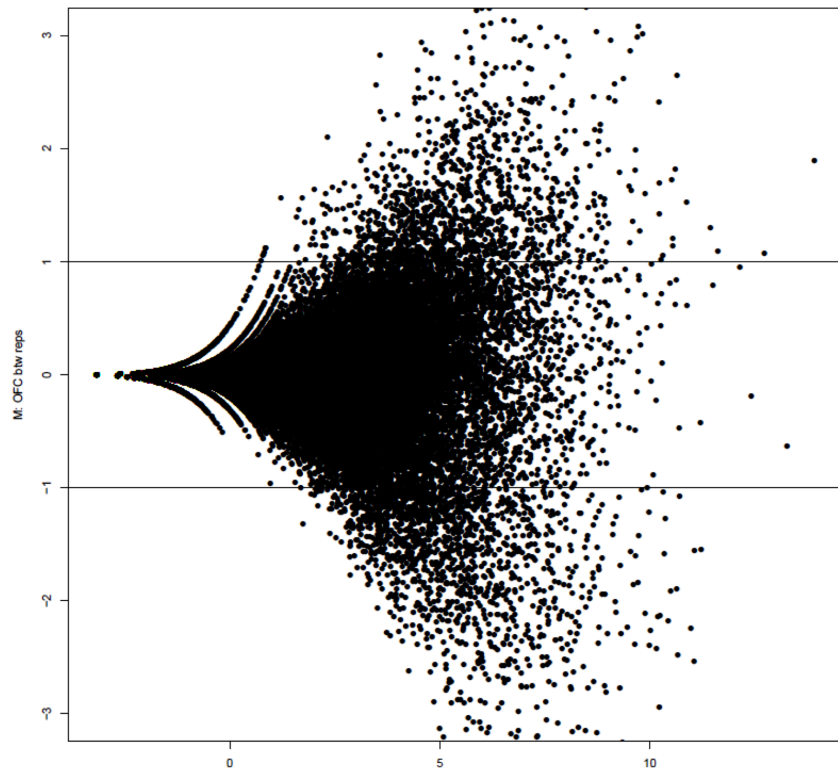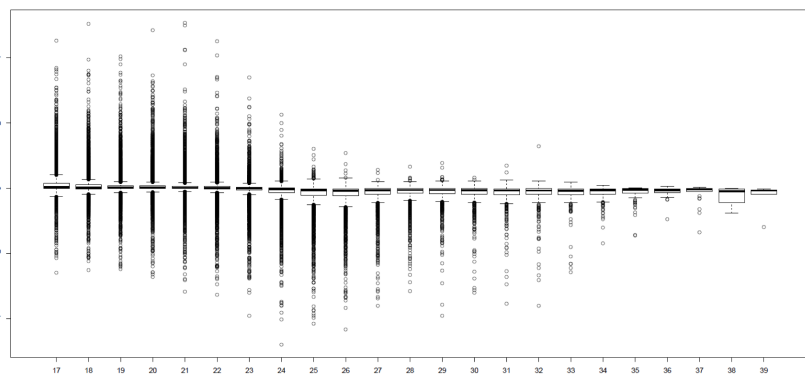

PV infected

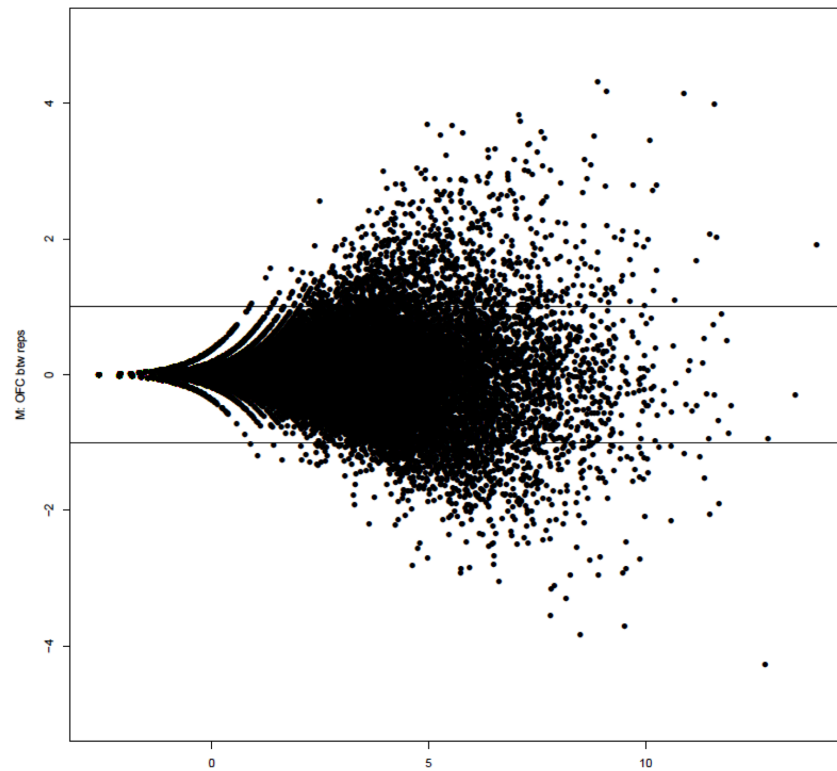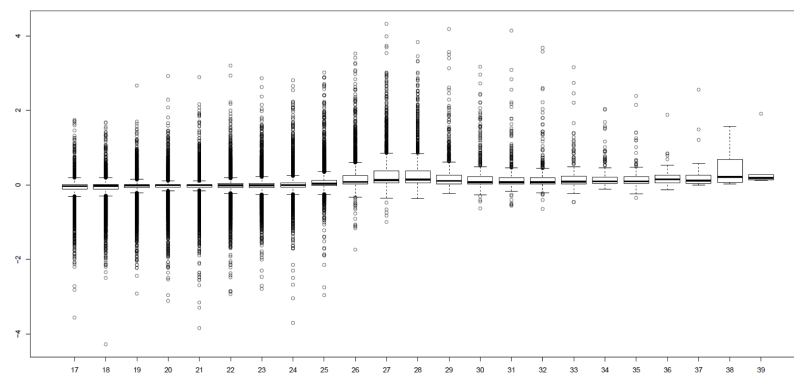

Supplement: Supplementary file 2 — Quantile normalisation. MA plots and distributions of DE (calculated using offset fold change method, OFC) separated per size class. In the MA plots, the average abundance (in log 2) is shown on the axis and the OFC (with offset = 20) is shown on the y axis. The CV, NK and PV correspond to Aspergillus fumigatus chrysovirus (AfuCV), a strain of Aspergillus fumigatus tetramycovirus-1 (AfuTmV-1) and Aspergillus fumigatus partitivirus-1 (AfuPV-1), respectively. (PDF 5547 kb) [file 12864_2017_3773_MOESM2_ESM.pdf]

**A.1**

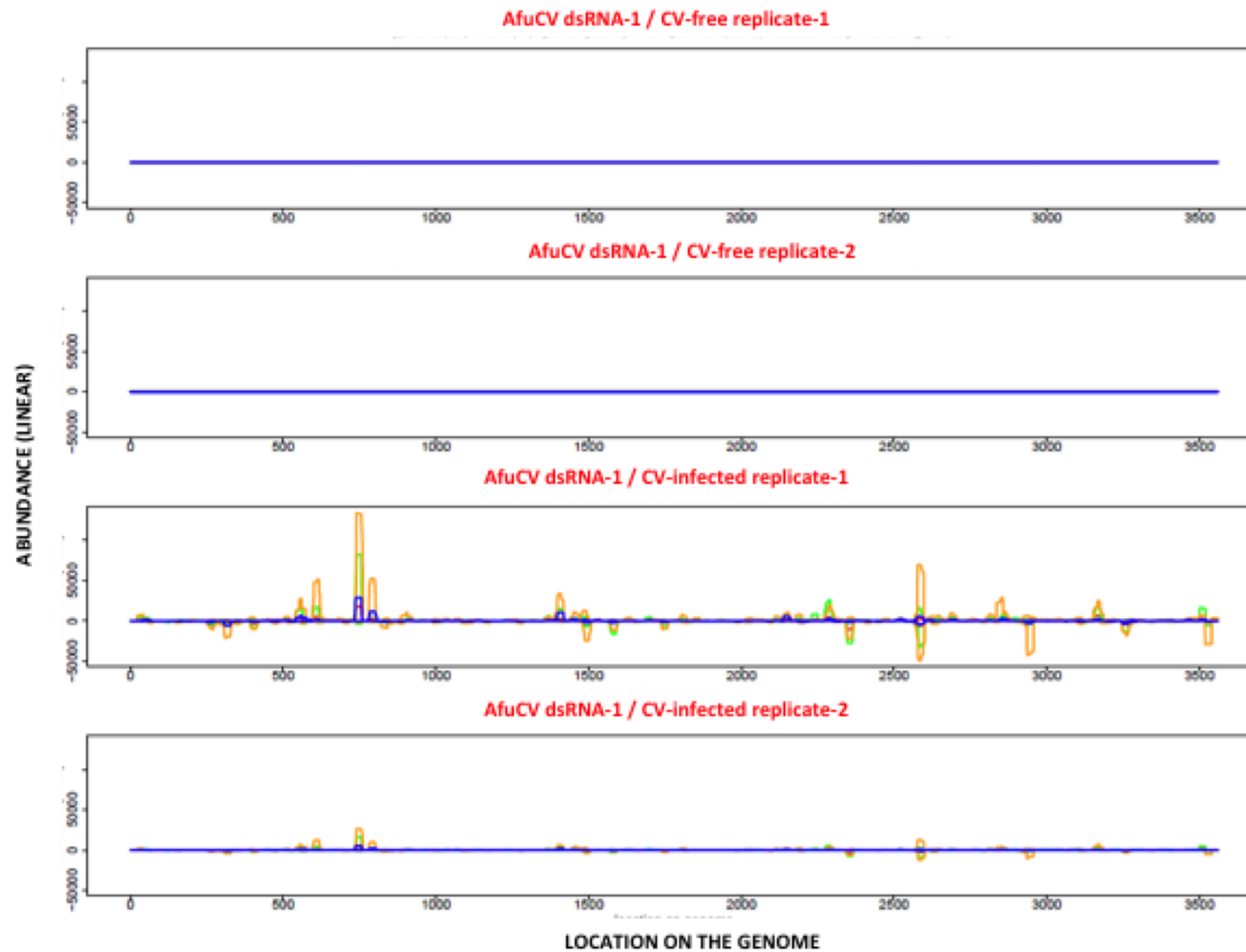

## A.2

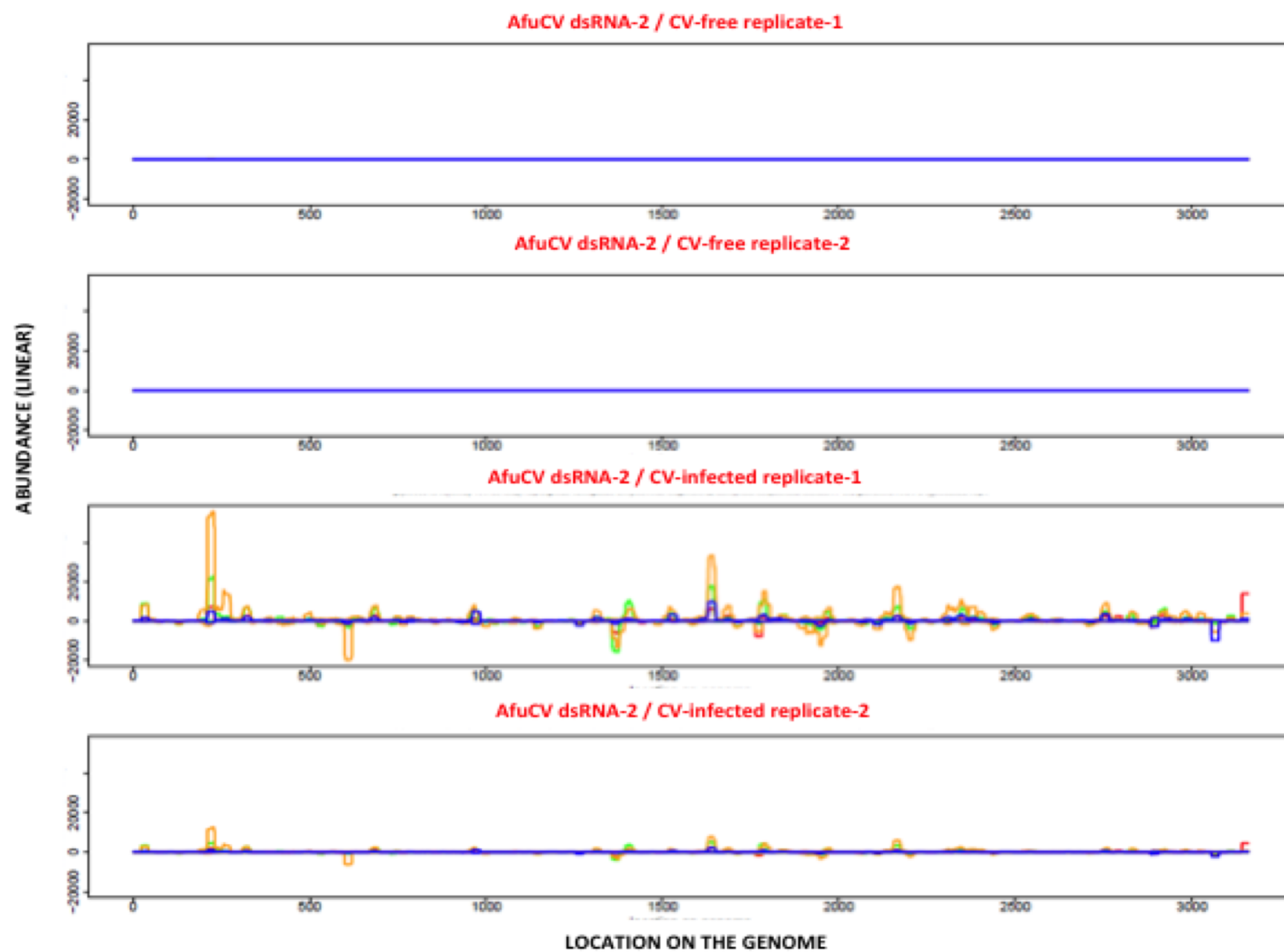

### A.3

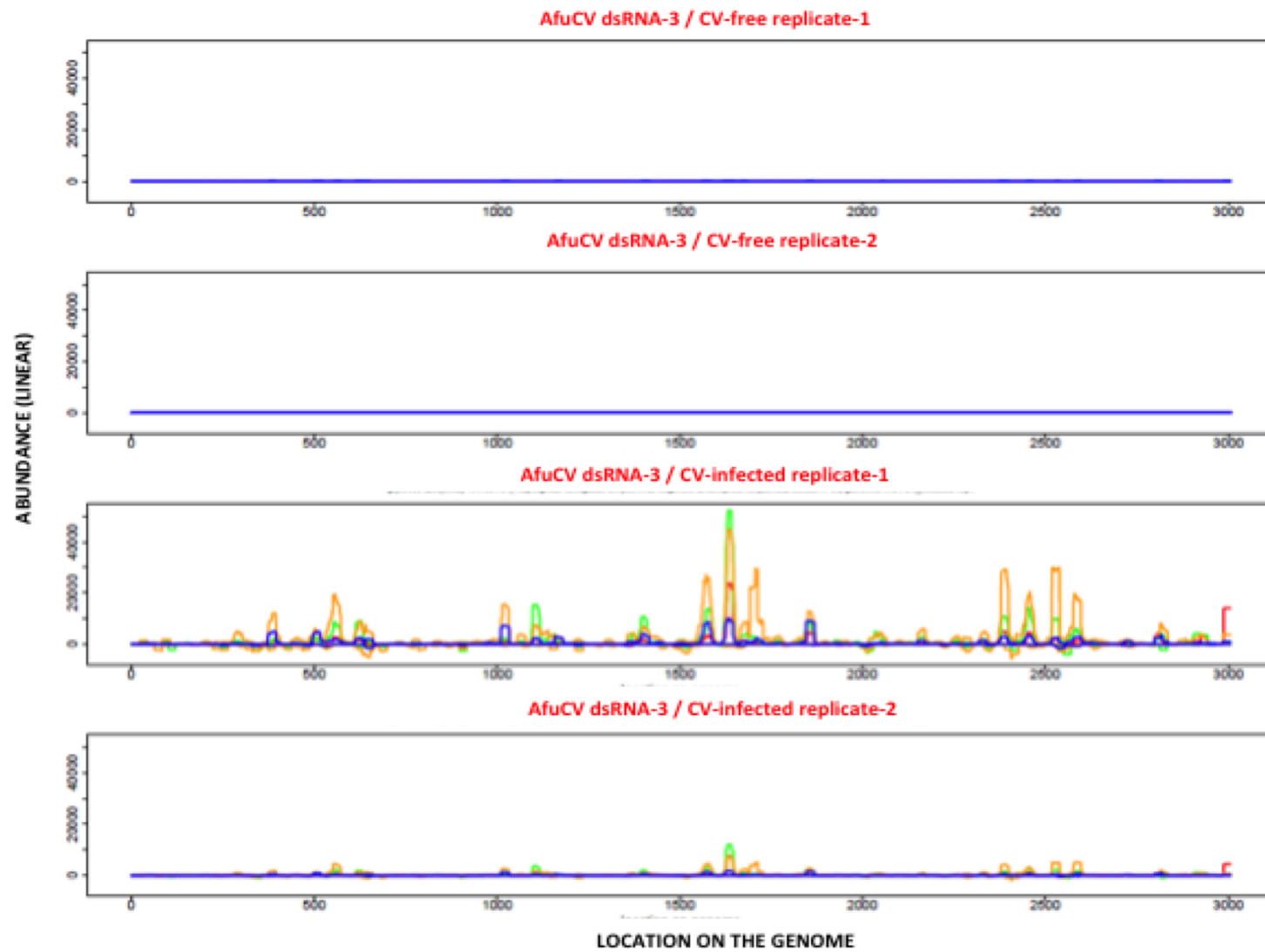

## A.4

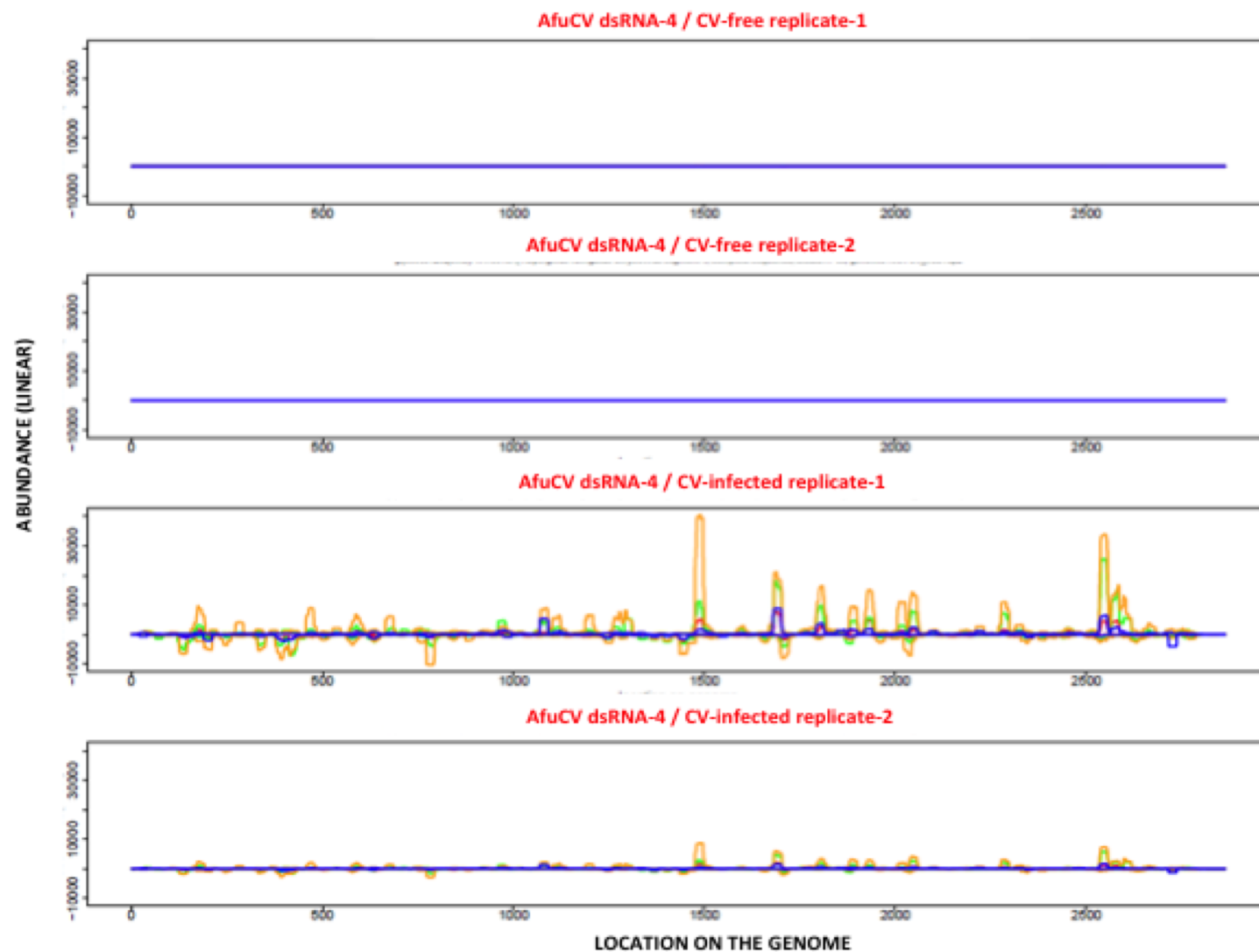

## B.1

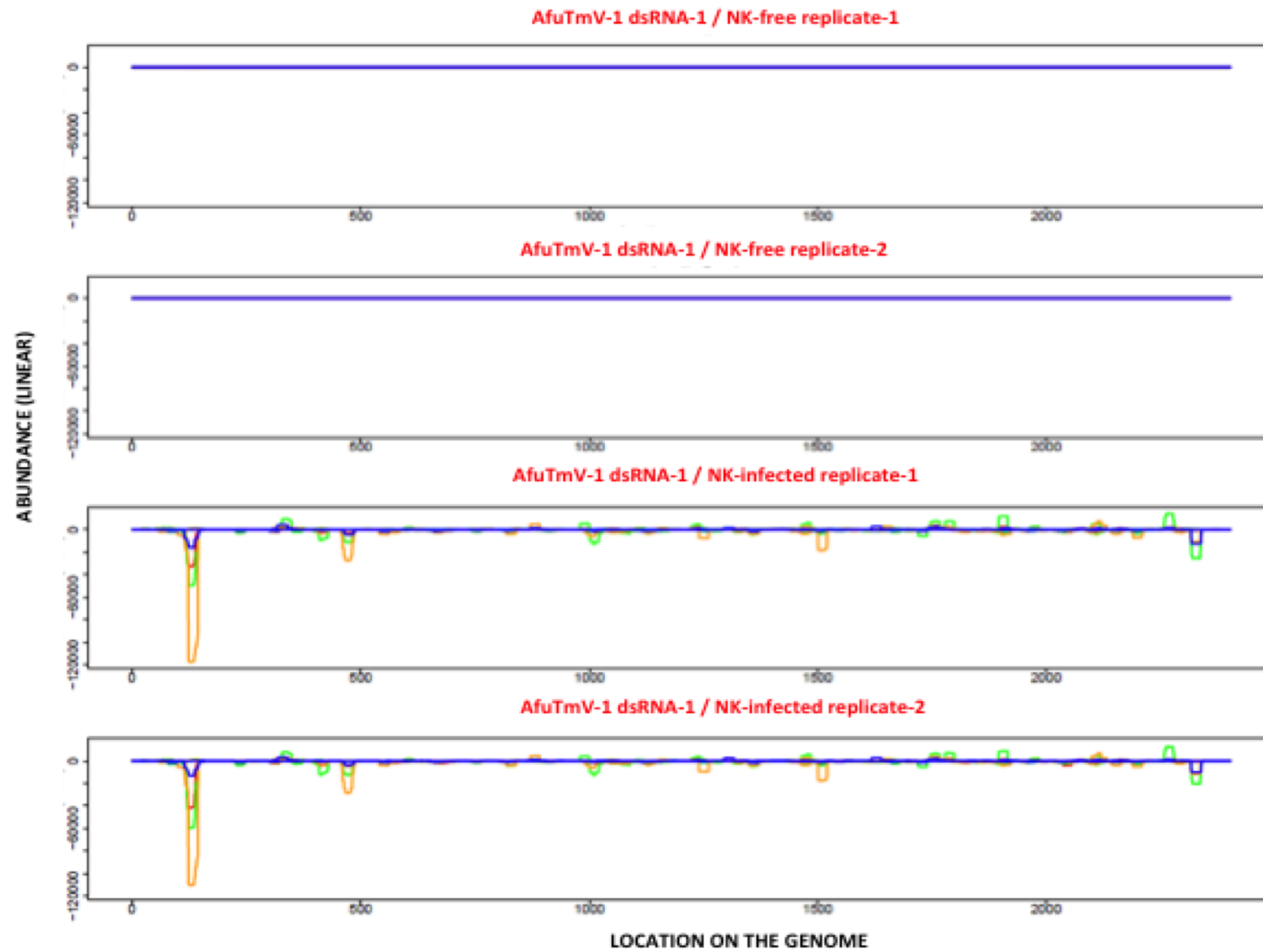

## B.2

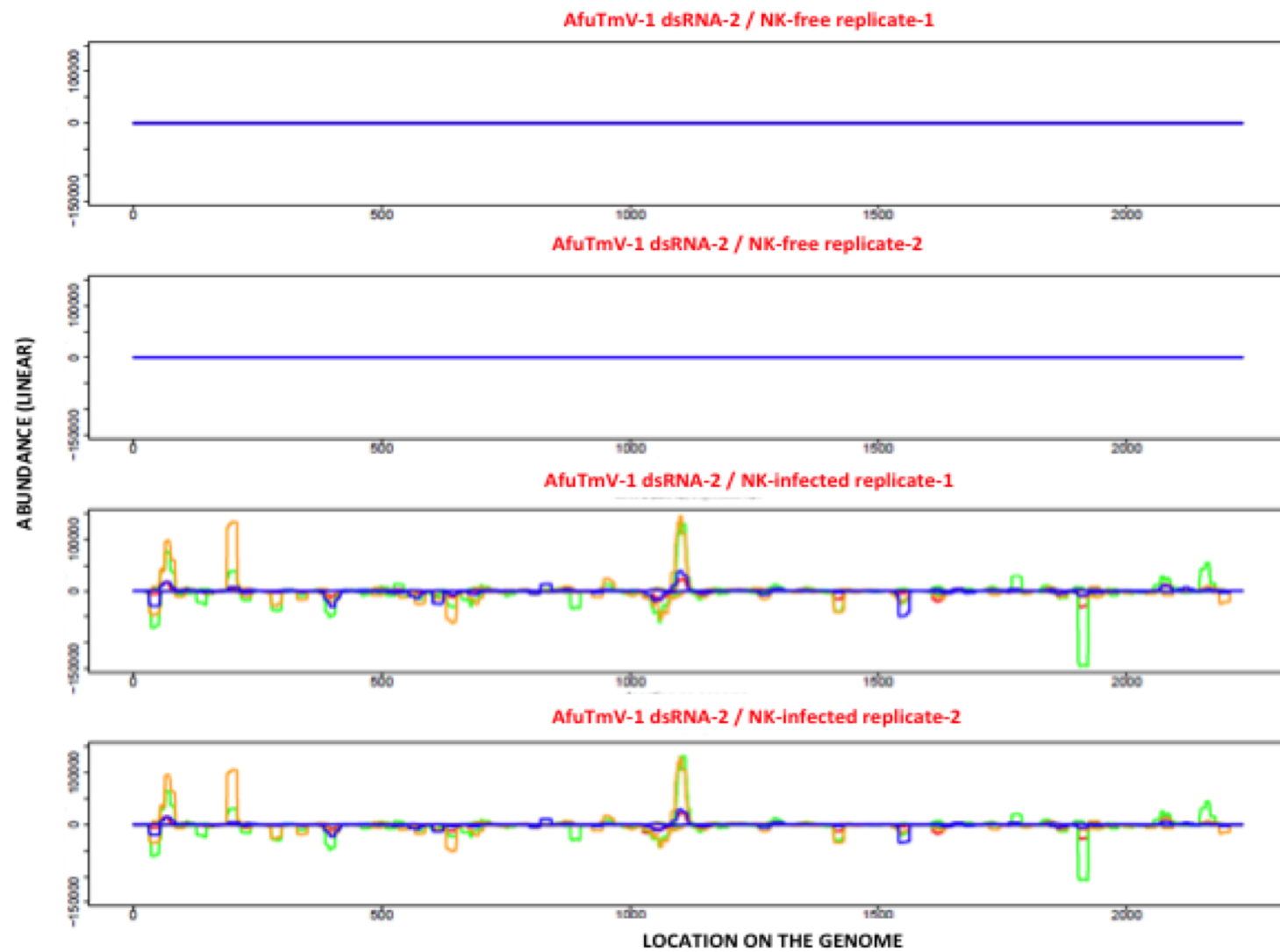

### B.3

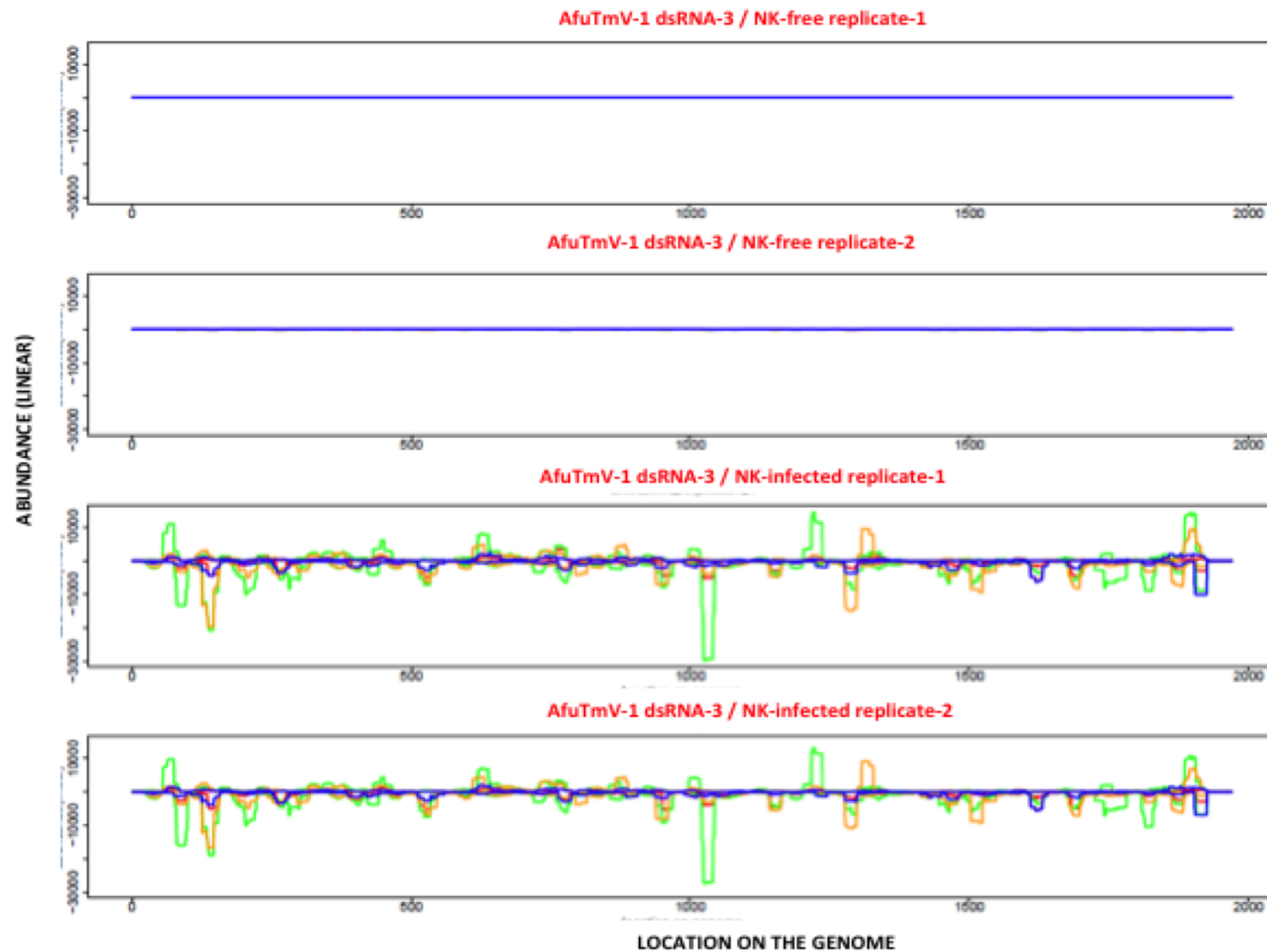

## B.4

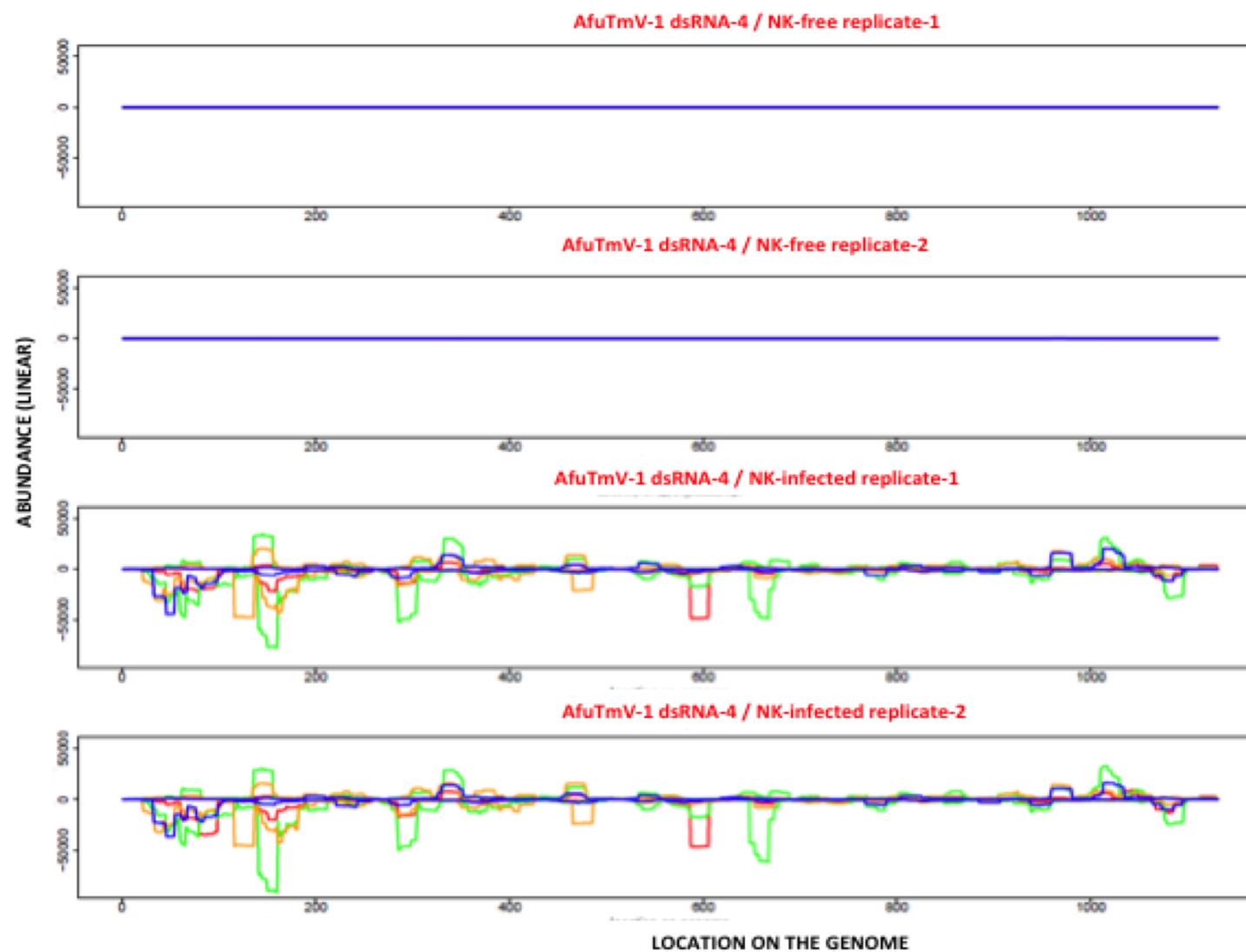

Supplement: Supplementary file 3 — Presence plots indicating the distribution and abundance of (A) Aspergillus fumigatus chrysovirus (AfuCV) and (B) Aspergillus fumigatus tetramycovirus-1 (AfuTmV-1)-derived sRNAs along the 4 segments of the related genomes. Double-stranded RNA segments are shown as 1, 2, 3 and 4 respectively. Colour code for the lines are red, green, orange and blue for 19 nt, 20 nt, 21 nt and 22 nt, respectively. Genomic coordinates were represented on the x axis. On the y axis, the point cumulative abundance of all incident reads (in linear scale) was represented. (PDF 8465 kb) [file 12864_2017_3773_MOESM3_ESM.pdf]
