# Supplementary material for: Profile and functional analysis of small RNAs derived from Aspergillus fumigatus infected with double-stranded RNA mycoviruses
Source: BMC Genomics. 2017 May 30;18:416. doi: 10.1186/s12864-017-3773-8 (PMC5450132; doi:10.1186/s12864-017-3773-8)
Supplement: Supplementary file 4 — List of probes designed to validate selected virus-derived sRNAs. The CV, NK and PV correspond to Aspergillus fumigatus chrysovirus (AfuCV), a strain of Aspergillus fumigatus tetramycovirus-1 (AfuTmV-1) and Aspergillus fumigatus partitivirus-1 (AfuPV-1), respectively. (PDF 37 kb) [file 12864_2017_3773_MOESM4_ESM.pdf]

Probe name	Location	sRNA sequence (5'→3')	Probe sequence (5'→3')
**U1-1 CONTROL**	-	TTCTTTGGCTCTATCCACTCTGTGGTGG	CCACCACAGAGTGGATAGAGCCAAAGAA
**CV_probe_1**	dsRNA1	TGACGTAGACATACCGAGAG	CTCTCGGTATGTCTACGTCA
**CV_probe_2**	dsRNA1	CAGAGTGTCTGGGGACGTACC	GGTACGTCCCCAGACACTCTG
**CV_probe_3**	dsRNA2	CGGTCCGATGGATCTGAGGCGTC	GACGCCTCAGATCCATCGGACCG
**CV_probe_4**	dsRNA2	GACGCCTCAGATCCATCGGACCG	CGGTCCGATGGATCTGAGGCGTC
**NK_probe_1**	dsRNA1	TGCACCCGTAGAGCTTGACG	CGTCAAGCTCTACGGGTGCA
**NK_probe_2**	dsRNA1	AGACAATGTCGGAGACGTAGG	CCTACGTCTCCGACATTGTCT
**NK_probe_3**	dsRNA2	TAGGGTGGCCCGGGACGAGC	GCTCGTCCCGGGCCACCCTA
**NK_probe_4**	dsRNA4	AGCGAGACGTCATCGACGGT	ACCGTCGATGACGTCTCGCT
**NK_probe_5**	NK-LINE	TGGTCGACGAACACGGATC	GATCCGTGTTCGTCGACCA
**PV_probe_1**	dsRNA1	ACTCTCACGCACCCAAGGAT	ATCCTTGGGTGCGTGAGAGT
**PV_probe_2**	dsRNA1	TCTGGACGTTGCGAAGGGTGA	TCACCCTTCGCAACGTCCAGA
**PV_probe_3**	dsRNA2	CTGGACAACTAGAGAGGCGGC	GCCGCCTCTCTAGTTGTCCAG
**PV_probe_4**	dsRNA2	GCCTTGAGATCCCACAGCGGAC	GTCCGCTGTGGGATCTCAAGGC
**PV_probe_5**	PV-LINE	TGCCTTGCCAGGCTTGGAC	GTCCAAGCCTGGCAAGGCA
