# Supplementary material for: Profile and functional analysis of small RNAs derived from Aspergillus fumigatus infected with double-stranded RNA mycoviruses
Source: BMC Genomics. 2017 May 30;18:416. doi: 10.1186/s12864-017-3773-8 (PMC5450132; doi:10.1186/s12864-017-3773-8)
Supplement: Supplementary file 5 — List of most abundant variants (with abundance greater than 100) which can hybridize with the PV probes. PV correspond to Aspergillus fumigatus partitivirus-1 (AfuPV-1). For each PV probe we present shorter and longer variant sequences, with up to 2 mis-matches relative to the probe. For each sequence we show the normalized abundance in the two infected replicates and the variant length. (PDF 38 kb) [file 12864_2017_3773_MOESM5_ESM.pdf]

	Probe	Sequence	Sequence Length (nt)	Abundance
PV_infected_1	PV_probe_1	ACTCTCACGCACCCAAGGAT	20	568
	PV_probe_1	TCCTTGGGTGCGTGAGAGT	19	439
	PV_probe_2	TCTGGACGTTGCGAAGGGTGA	21	164899
	PV_probe_2	TGGACGTTGCGAAGGGTGA	19	25248
	PV_probe_2	TCTGGACGTTGCGAAGGGT	19	16102
	PV_probe_2	TCTGGACGTTGCGAAGGGTG	20	14785
	PV_probe_2	TCTGGACGTTGCGAAGGGTGC	21	11010
	PV_probe_3	CTGGACAACTAGAGAGGCGGC	21	134509
	PV_probe_3	TGGACAACTAGAGAGGCGGC	20	37337
	PV_probe_3	CTGGACAACTAGAGAGGCGG	20	36302
	PV_probe_3	CTGGACAACTAGAGAGGCGGA	21	19210
	PV_probe_4	TCCGCTGTGGGATCTCAAGGC	21	6286
	PV_probe_4	GCCTTGAGATCCCACAGCGGAC	22	3333
	PV_probe_5	TGCCTTGCCAGGCTTGGAC	19	1264
PV_infected_2	PV_probe_1	ACTCTCACGCACCCAAGGAT	20	417
	PV_probe_1	TCCTTGGGTGCGTGAGAGT	19	190
	PV_probe_2	TCTGGACGTTGCGAAGGGTGA	21	90694
	PV_probe_2	TGGACGTTGCGAAGGGTGA	19	14112
	PV_probe_2	TCTGGACGTTGCGAAGGGTG	20	7452
	PV_probe_2	TCTGGACGTTGCGAAGGGT	19	6782
	PV_probe_2	TCTGGACGTTGCGAAGGG	18	1827
	PV_probe_3	CTGGACAACTAGAGAGGCGGC	21	43360
	PV_probe_3	TGGACAACTAGAGAGGCGGC	20	12627
	PV_probe_3	CTGGACAACTAGAGAGGCGG	20	7817
	PV_probe_3	CTGGACAACTAGAGAGGCGGA	21	5433
	PV_probe_4	TCCGCTGTGGGATCTCAAGGC	21	2418
	PV_probe_4	GCCTTGAGATCCCACAGCGGAC	22	1309
	PV_probe_5	TGCCTTGCCAGGCTTGGAC	19	495
