# Supplementary material for: Profile and functional analysis of small RNAs derived from Aspergillus fumigatus infected with double-stranded RNA mycoviruses
Source: BMC Genomics. 2017 May 30;18:416. doi: 10.1186/s12864-017-3773-8 (PMC5450132; doi:10.1186/s12864-017-3773-8)
Supplement: Supplementary file 6 — Characteristics of miRNA-like candidates in A. fumigatus. For each miRNA-like candidate we present the hairpin sequence, the mature sequence, incident annotation based on version Af293, version s03-m04-r22 of the A. fumigatus genome, the samples for which the candidate is differentially expressed and the corresponding secondary structure. (PDF 217 kb) [file 12864_2017_3773_MOESM6_ESM.pdf]

Name	Hairpin sequence	Mature sequence	Probe sequence (5'→ 3')	Annotation	Differentially expressed in	Folding
**miRCat_2**	CGCGGUCCUUCCAC<br>GCCUUCCAGACCAGC<br>GAGCGGGCCCGCGA<br>GGUCGAGACGGGAA<br>ACCGCUUGGUGGAG<br>AGGCCCAGGAAGCG<br>GGUGAUGGCGUUGG<br>AGCUGAGACUGUCG	TGGCGTTGGAGCTGAGACTG	CAGTCTCAGCTCCAACGCCA	No annotation	CV infected	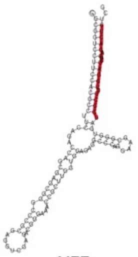<br>MFE:<br>-51.60
**FOLDED-2**	GUCAGAGCCUUUGA<br>GGUUGUCUCCCUGA<br>CAUGGUACUUGAAG<br>CUGAGCUUCUUGUC<br>AAAGAGGACCCCCAG<br>CCAACGCAGGUAAG<br>GGCGAGUUGUAUUC<br>UCGGAGACUGUGAC	GAGTTGTATTCTCGGAGACTGTGAC	GTCACAGTCTCCGAGAATACAACTC	LINEs	CV, NK, PV infected	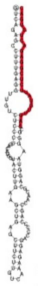<br>MFE:<br>-36.80
