# Supplementary material for: Profile and functional analysis of small RNAs derived from Aspergillus fumigatus infected with double-stranded RNA mycoviruses
Source: BMC Genomics. 2017 May 30;18:416. doi: 10.1186/s12864-017-3773-8 (PMC5450132; doi:10.1186/s12864-017-3773-8)
Supplement: Supplementary file 7 — Characteristics of Folded-1 (PDF 787 kb) [file 12864_2017_3773_MOESM7_ESM.pdf]

Candidate Folded-1 was identified as differentially expressed under PV and NK infections (Figure SF1); in addition, a secondary structure with an adjusted minimum free energy (aMFE) of -32.20 was predicted. The putative hairpin and mature sequence are presented in table ST1. The "mature" sequence was validated using northern blotting (Figure SF2). The low throughput validation confirmed the presence of one abundant variant (sharp band on the northern blot) and confirmed the size. Subsequent advances in the annotation of *A. fumigatus* isolates, but not in the *A. fumigatus* 293 strain, indicate that it is likely that folded1 is an rRNA fragment.

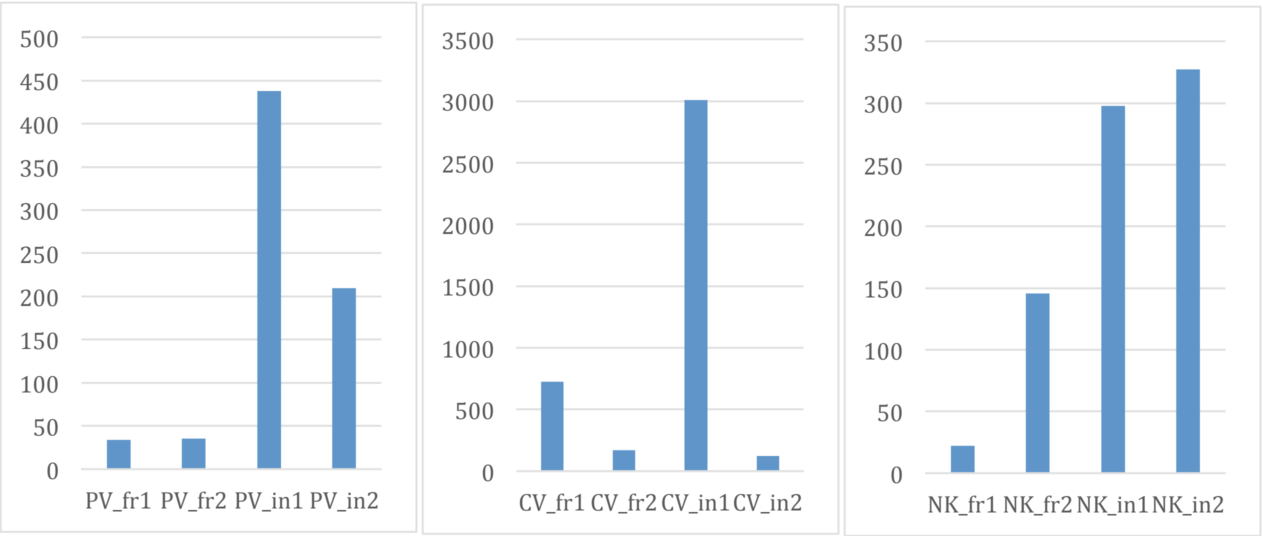

Figure SF1. Normalized expression levels of Folded-1 in the PV, CV and NK virus-free and virus-infected samples. On the y axis we represent the normalized expression levels, in linear scale; on the x-axis we represent the samples.

Name	Hairpin sequence	Mature sequence	Probe sequence (5'→ 3')	Differentially expressed in	Folding
FOLDED-1	ACCCGGTCATTTAGAGGAAGTAAAAGTCGTAACAAGGTTTCCGTAGGTGAACCTGCGGAAGGATCATTACCGAGTGAGGGCCCTCTGGGTCCAACC	CCGAGTGAGGGCCCTCTGGGTCCAACC	GGTTGGACCCAGAGGGCCCTCACTCGG	CV, NK, PV infected	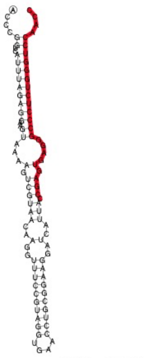 MFE: -32.30

Table ST1. Characteristics of the Folded-1 sequence such as the hairpin and putative "mature" sequence and the secondary structure corresponding to the minimum free energy. On the secondary structure, we indicate the location of the "mature" sequence in red.

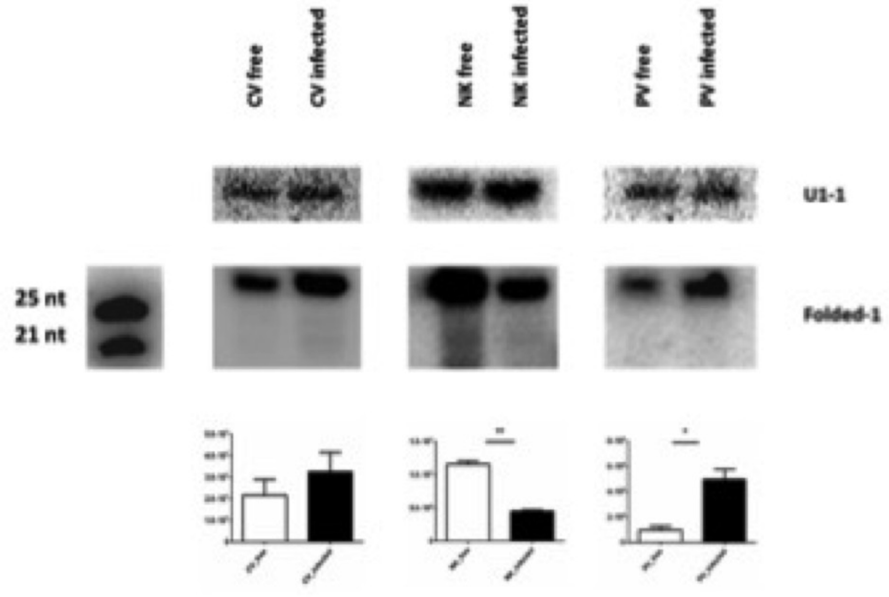

Figure SF2. Quantitative northern blot analysis of Folded-1. The CV, NK and PV correspond to Aspergillus fumigatus chrysovirus (AfuCV), a strain of Aspergillus fumigatus tetramycovirus-1 (AfuTmV-1) and Aspergillus fumigatus partitivirus-1 (AfuPV-1), respectively. Individual lanes loaded with 4 µg sRNA were blotted and onto membranes and hybridised at 37°C overnight with. P^{32} labelled oligonucleotides designed to detect miRNA-like candidate species. *A. fumigatus* U1-1 small nuclear RNA was used as a loading control throughout and the miRNA bands were normalized using the integrity of the U1-1 bands. Relative expression levels were plotted using the GraphPad Prism 6.0 software and *P* values were estimated using an unpaired t-test with Welch's correction. Error bars were calculated using the standard error values. *P* values less than 0.05 were accepted as statistically significantly different and are shown with an asterisk (** indicates P ≤ 0.01; n=3).

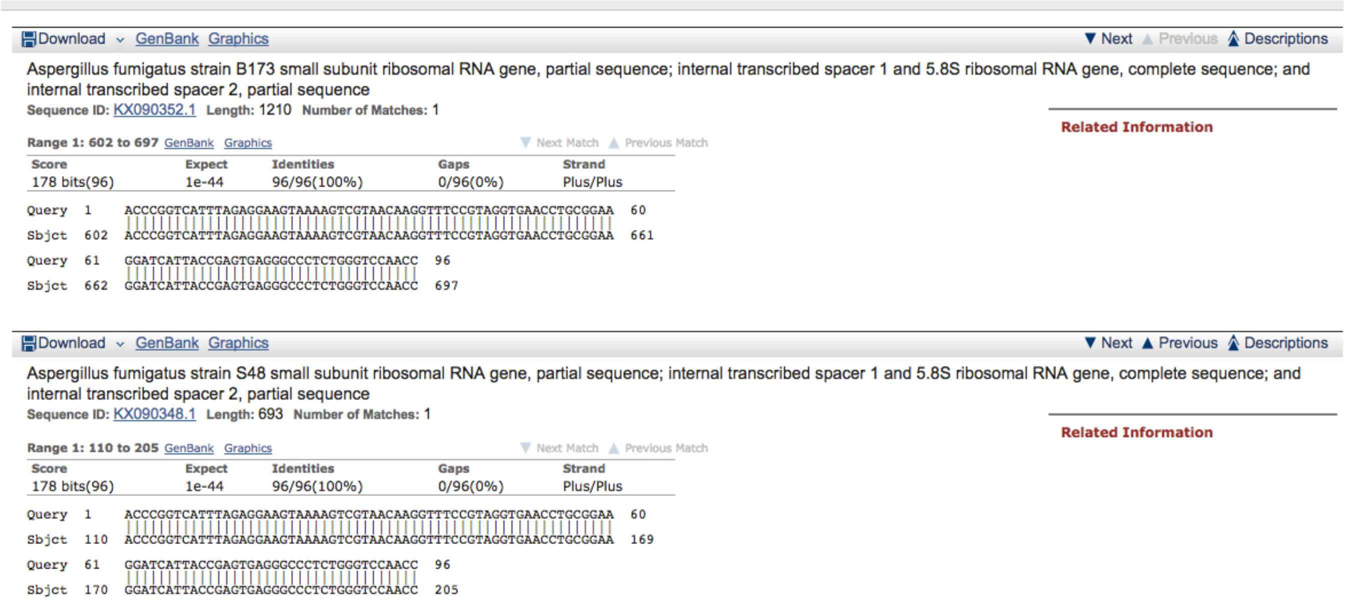

Figure SF3. BLAST result indicating that the folded-1 sequence is similar (plus/plus) with an internal transcribed spacer for the 5.8S ribosomal RNA gene in *A. fumigatus*, strain B173 and strain S48 (annotated in December 2016).

Based on this evidence, we conclude that Folded-1 is highly likely an rRNA-derived read which is DE under virus infection.
