# Supplementary material for: Profile and functional analysis of small RNAs derived from Aspergillus fumigatus infected with double-stranded RNA mycoviruses
Source: BMC Genomics. 2017 May 30;18:416. doi: 10.1186/s12864-017-3773-8 (PMC5450132; doi:10.1186/s12864-017-3773-8)
Supplement: Supplementary file 8 — Histograms showing the differentially expressed (−2 ≥ DE or DE ≥ 2) sRNA loci and annotations. The sRNA loci of all three viruses were identified and expressions plotted for CV (A), NK (B) and PV (C). The CV, NK and PV correspond to Aspergillus fumigatus chrysovirus (AfuCV), a strain of Aspergillus fumigatus tetramycovirus-1 (AfuTmV-1) and Aspergillus fumigatus partitivirus-1 (AfuPV-1), respectively. Annotations of the regions of putative sources of sRNAs were presented in red to indicate those differentially expressed in virus-infected cases and in blue to indicate the ones differentially expressed in virus-free examples. Comparisons were made for all three combinations (1_1: virus_free_rep1 and virus_infected_rep_1; 1_2: virus_free_rep1 and virus_infected_rep_2; 2_1: virus_free_rep2 and virus_infected_rep_1; 2_2: virus_free_rep2 and virus_infected_rep_2). (PDF 1446 kb) [file 12864_2017_3773_MOESM8_ESM.pdf]

## A

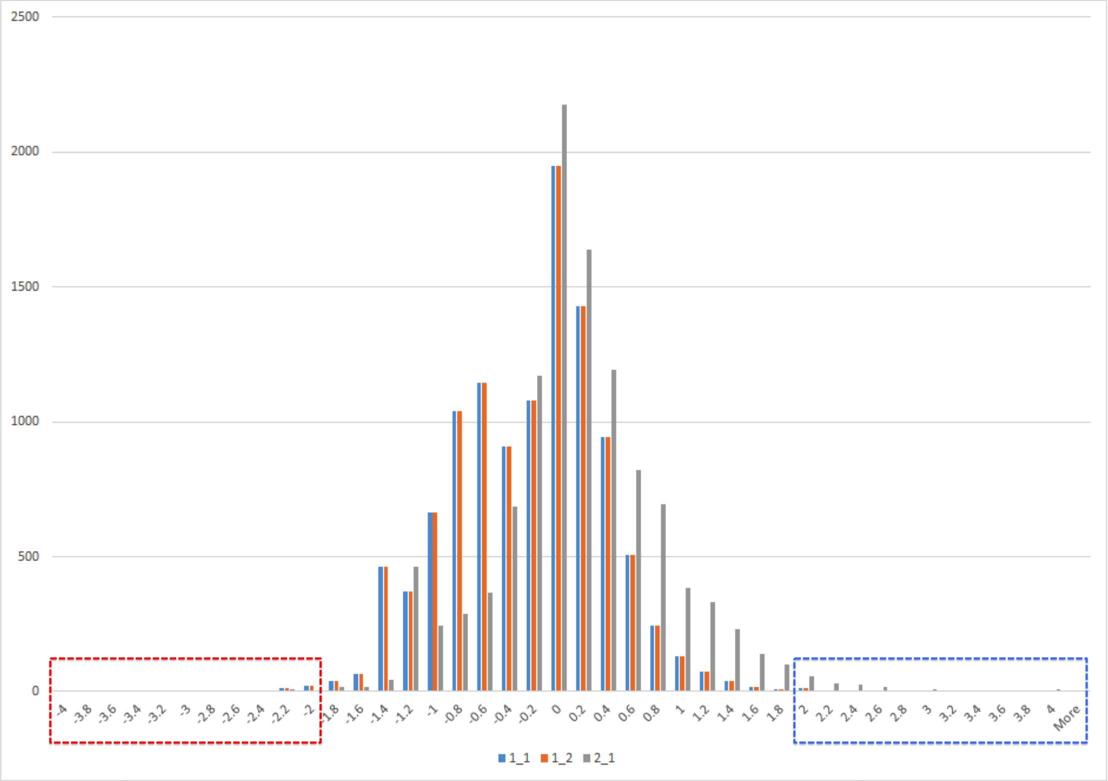


Afu2g13260
Afu3g3330
Afu2g07670
Afu3g03420, *sidD*
Afu3g08010, *ace1*

Afu5g00730
Afu5g02330, *aspf1*
Afu5g07600
Afu8g06070

## B

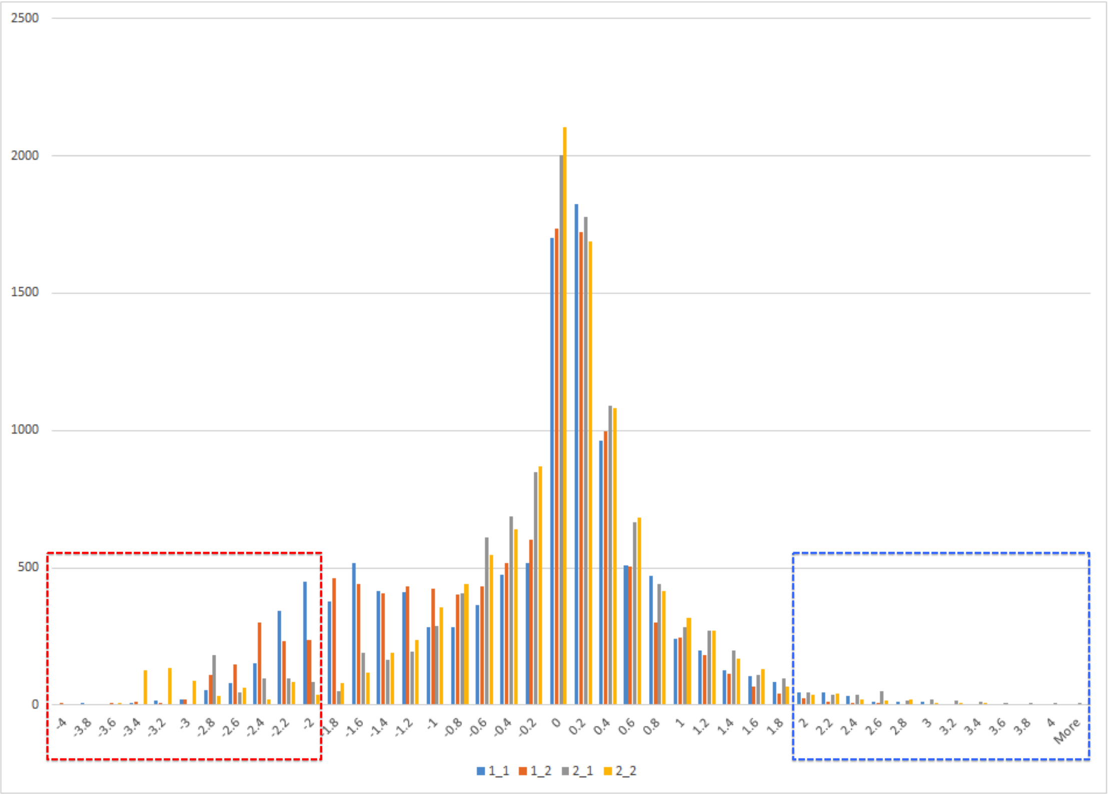


Afu1g14540
Afu2g05830
Afu3g11960
Afu5g12130, *rab7*
[illegible]

Afu3g07870
Afu4g11250, *cafA*
Afu5g12730, *nrps8*

**C**

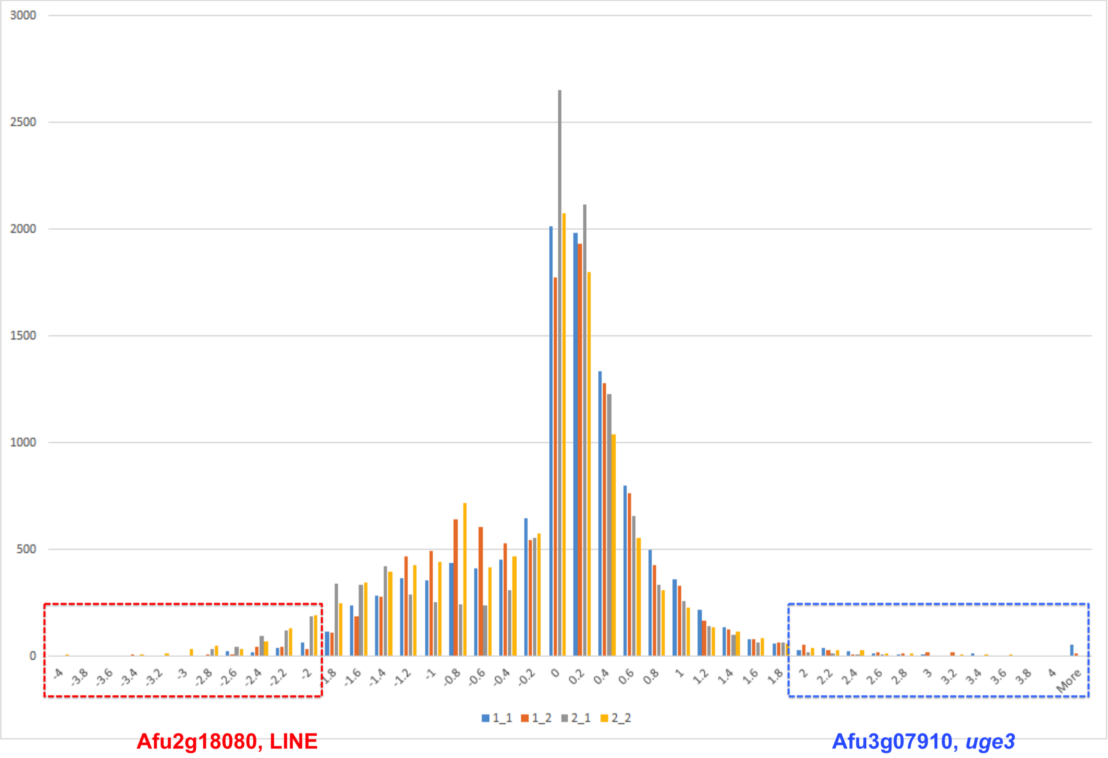


**Afu2g18080, LINE**
**Afu5g00270, LINE**
**Afu5g06560**
**Afu8g01040, LINE**
**Afu8g00310, LINE**
**Afu8g04290, LINE**
**Afu8g01040, LINE**

**Afu3g07910, *uge3***
**Afu4g08370**
**Afu6g07770**
**Afu8g06070**
