# Supplementary material for: Profile and functional analysis of small RNAs derived from Aspergillus fumigatus infected with double-stranded RNA mycoviruses
Source: BMC Genomics. 2017 May 30;18:416. doi: 10.1186/s12864-017-3773-8 (PMC5450132; doi:10.1186/s12864-017-3773-8)
Supplement: Supplementary file 9 — qPCR validations of RNAi genes and genes responding to the virus infection. (PDF 501 kb) [file 12864_2017_3773_MOESM9_ESM.pdf]

Section 1. qPCR evaluation of expression levels in virus free and virus infected *A. fumigatus* isolates of RNAi genes: Dicer like-1, Dicer like-2, dsRNA-specific RNase-III, Argonaute, QDE-2, histone deacetylase, RNA-directed RNA polymerase-1 and RNA-directed RNA polymerase-2.

The GenBank accession numbers and primer sequences are listed in table S1. The reference gene for the normalisation of expression levels was β-tubulin. The CV, NK and PV correspond to Aspergillus fumigatus chrysovirus (AfuCV), a strain of Aspergillus fumigatus tetramycovirus-1 (AfuTmV-1) and Aspergillus fumigatus partitivirus-1 (AfuPV-1), respectively. Quantitative PCR (qPCR) assays were performed using SYBR Green I (Invitrogen) as a fluorescent dye and monitored using the ABI Prism 7700 system (Perkin-Elmer Applied Biosystems). Three biological replicates for eight isolates were carried out in duplicate in MicroAmp 96-well plates (Applied Biosystems, USA). cDNA concentration of the qPCR reaction was standardised as approximately 100 ng for each sample. In all experiments appropriate negative controls containing no template (NTC) and no reverse transcriptase enzyme (-RT) were subjected to the same procedure to detect any possible contamination. Amplicons were also electrophoresed on agarose gel to check amplicon size and qRT-PCR process. The level of mRNA expression was measured in different isolates of *A. fumigatus* by relative quantification using the comparative $2^{-\Delta Ct}$ method (Livak & Schmittgen, 2001). Relative expression levels were plotted using GraphPad Prism 6.0 programme and *P*-values were calculated using two-tailed Student’s t-test. Error bars were calculated by using the standard deviation values. *P*-values less than 0.05 were accepted as statistically significant difference and illustrated by asterisk (*, **, ***, **** indicate P-value ≤ 0.05, 0.01, 0.001 and 0.0001, respectively; n=3).

Gene	GenBank Accession number	Abbreviations	Primer Sequence
β-tubulin<br>(Balajee et al., 2005)	AFUA_1G10910	β-tubulin-F<br>β-tubulin-R	5′AATTGGTGCCGCTTTCTGG3′<br>5′AGTTGTCGGGACGGAATAG3′
Dicer like-1<br>(RNA helicase/RNase III)	AFUA_5G11790	Dicer-1-F<br>Dicer-1-R	5′AATATTATGCGGCGCTTCTG3′<br>5′TGCAGAGAACTGGCATAACG3′
Dicer like-2<br>(RNA helicase/RNase III)	AFUA_4G02930	Dicer-2-F<br>Dicer-2-R	5′TTTGATGAAGCGCACCACT3′<br>5′CAAGAGCTCCTGGTGATTCG3′
dsRNA specific ribonuclease-III	AFUA_5G06830	Ribo-III-F<br>Ribo-III-R	5′CGCAAGAGCAGGTATTGAGG3′<br>5′CGGTTCGATACCAAATGTCC3′
Argonaute<br>(eukaryotic translation initiation factor eIF-2C4)	AFUA_3G11010	Argo-F<br>Argo-R	5′GCTTGCCTGGTCCATGAATA3′<br>5′CATAGAGGTCCGCCCAGTAA3′
RNA interference and gene silencing protein (QDE-2)	AFUA_8G05280	QDE-2-F<br>QDE-2-R	5′ACCAGGGAGAACCCATCCTA3′<br>5′GATTCTTGAGGCCGAGGAC3′
Histone deacetylase	AFUA_2G03390	His-F<br>His-R	5′CCTCCGATTGACCTGAATCTC3′<br>5′CCAGGCTATGTCATCCTG3′
RNA-directed RNA polymerase-1	AFUA_5G09430	RdRP-1-F<br>RdRP-1-R	5′AGGGACGAATCATGGAACAG3′<br>5′GCCTTCAATCCACTTCCAAA3′
RNA-directed RNA polymerase-2 (Sad-1)	AFUA_3G06790	RdRP-2-F<br>RdRP-2-R	5′AACCACCCCCTCAGAAAGAT3′<br>5′GGTAGTTTCACCTCAGCAGGA3′

Table ST1. ***A. fumigatus*** **RNAi genes validated using qPCR assays; the β-tubulin gene was used as reference gene.** For each gene, we present its common name, the GenBank accession number, its abbreviation and the forward (first row) and reverse (second row) primers used for the qPCR quantification.

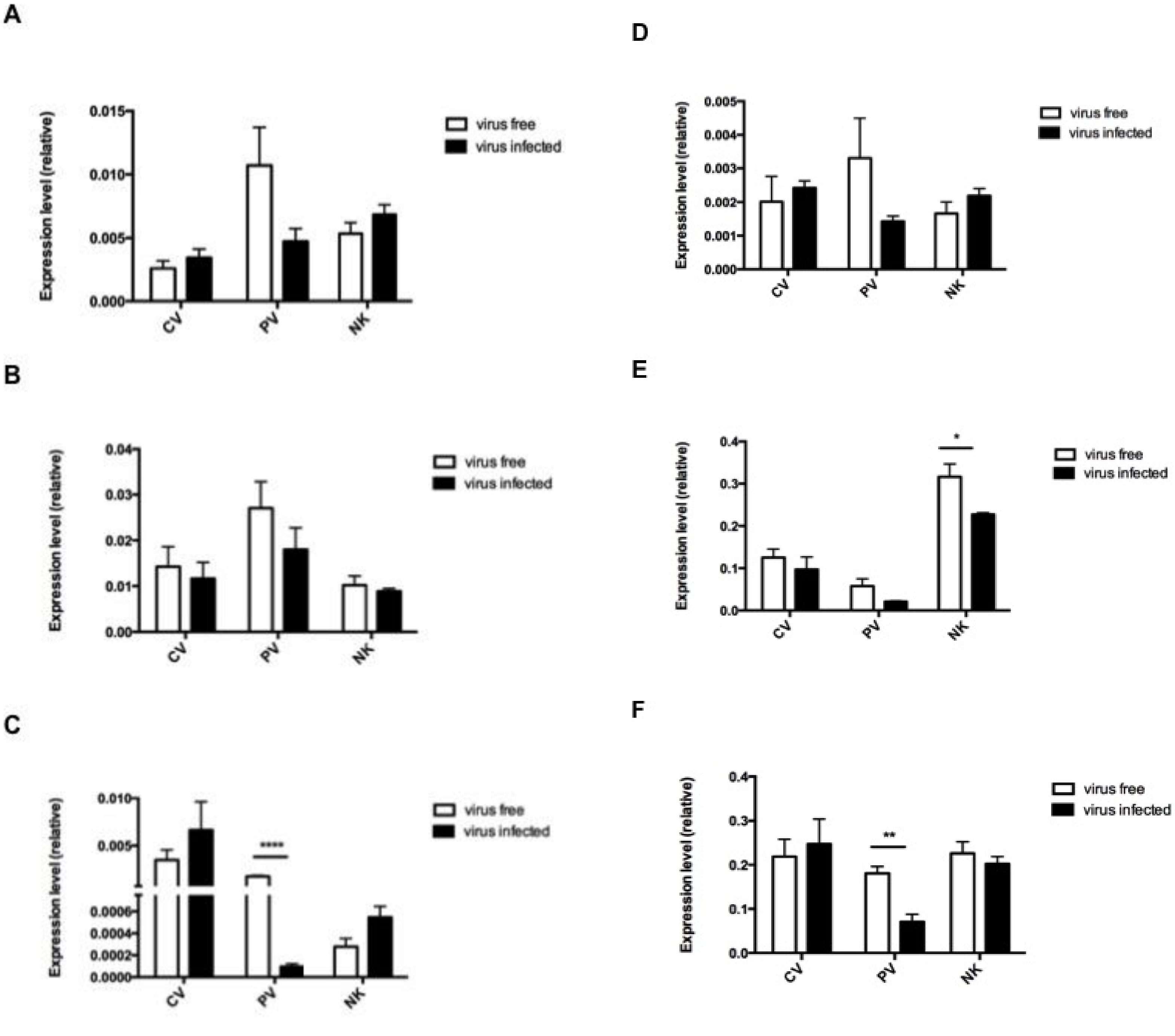
A
B
C
D
E
F
Expression level (relative)
virus free
virus infected
CV
PV
NK
0.000
0.005
0.010
0.015
0.00
0.01
0.02
0.03
0.04
0.0000
0.0002
0.0004
0.0006
0.001
0.002
0.003
0.004
0.005
0.0
0.1
0.2
0.3
0.4
****
*
**

G

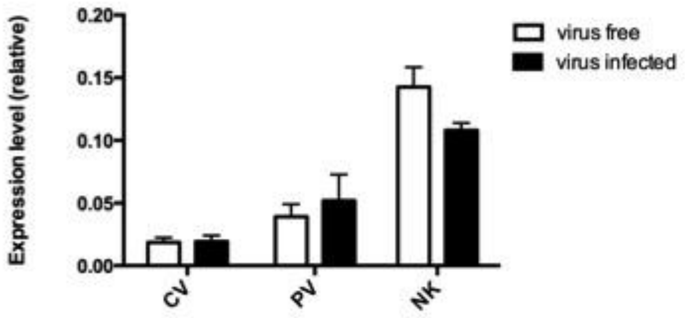

H

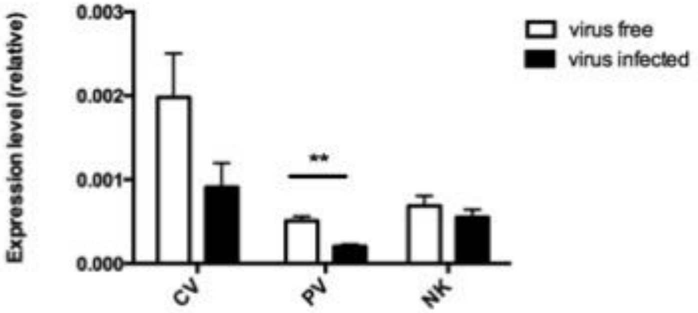

Figure SF1. qPCR validations for RNAi genes: Dicer like-1 (panel A), **Dicer like-2** (panel B), **dsRNA-specific RNase-III** (panel C), **Argonaute** (panel D) and **QDE-2** (panel E), **histone deacetylase** (panel F), **RNA-directed RNA polymerase-1** (panel G) and **RNA-directed RNA polymerase-2** (panel H). The qPCR was done on three biological replicates, each with two technical replicates. The values for the biological replicates are the averages of the measurements of the technical replicates. The error bars are based on the standard deviation of the biological replicates values; the test used for determining whether the difference between the non-infected and the infected samples was significant is the two-tailed Student's t-test. Genes dsRNA-specific RNase-III, histone deacetylase and RNA-directed RNA polymerase-2 were significantly down regulated in the PV infection. QDE-2 gene was significantly down regulated in the NK infection.

Section 2.

qPCR evaluation of putative targets of the viral siRNAs derived from the *A. fumigatus* chrysovirus and the partitivirus-1

To identify potential targets of virus-derived siRNAs (vsiRNAs), reads mapping to both the viral and fungal genome were further investigated. The reads incident to the viral genome with zero mismatches and also incident with the fungal genome with up to 2 mismatches led to the identification, as putative targets of important, pathogenicity–related genes. Two candidates were selected; their expression levels were measured using qPCRs in the presence and absence of virus infection.

The virus-derived siRNAs, their potential targets and primers used to amplify putative target genes are listed in table ST2. Nucleotides in red and underlined indicate the location of the mismatches. Relative expression levels were plotted using GraphPad Prism 6.0 programme and *P*-values were estimated using two-tailed Student's t-test with unequal variance. Error bars were calculated using the standard error values. *P*-values less than 0.05 were accepted as statistically significant difference and illustrated by an asterisk (* mean $P \leq 0.05$; n=3).

sRNA seq (5’ → 3’)	Target gene name and accession number	Primer name	Primer Sequence
TTGGTGAACATCTTCCGGT	*pksP/alb1* AFUA_2G17600	pksP-F<br>pksP-R	5’TGTCCATATTGCCTTCAAGC3’<br>5’GCATCTTCCATCAGGAGAGC3’
ACCCGTGGAGAAGACCAC	*pyrG* AFUA_2G08360	pyrG-F<br>pyrG-R	5’CTTCGTTATGGGTTTCGTGTC3’<br>5’CCGATGCAGGAGTCTGGTAT3’

Table ST2. Putative host, *A. fumigatus*, targets of vsiRNAs. Sequence TTGGTGAACATCTTCCGGT matches with 0 mismatches on the CV virus; it also matches (plus/minus) to the *pksP* gene; the sequence ACCCGTGGAGAAGACCAC matches to the PV and with 2 mis-matches on the plus/minus against the *pvrG* gene; the nucleotides highlighted in red indicate the location of the mis-matches. The forward and reverse primers used for the evaluation of the gene expressions are presented in the last column.

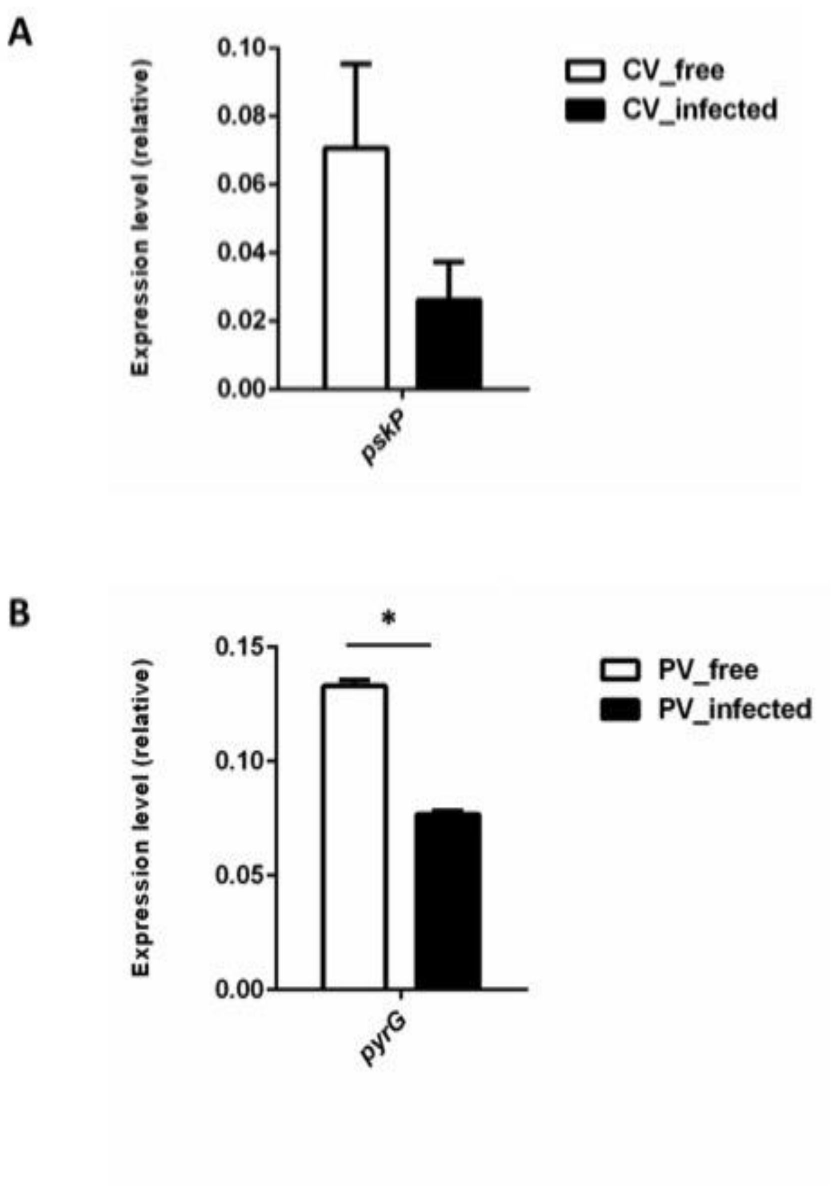

Figure SF2. qPCR validation of putative targets of vsiRNAs. The pskP gene (panel A) is a putative target of a fragment incident with the CV, the pyrG gene (panel B) is a putative target of a fragment incident with the PV. The error bars indicate the standard deviation; the test used for determining whether the difference between the non-infected and the infected samples was significant is the two-tailed Student's t-test. The pyrG is significantly different between the virus free and virus infected samples. The pskP gene is only marginally differentially expressed.

References:

Balajee SA, Gribskov JL, Hanley E, Nickle D, Marr KA. *Aspergillus lentulus* sp. nov., a new sibling species of *A. fumigatus*. Eukaryot Cell 2005; 4: 625-632.

Livak KJ, Schmittgen TD. Analysis of relative gene expression data using real-time quantitative PCR and the 2(-Delta Delta C(T)) Method. Methods 2001; 25: 402-408.
